# Single-cell and spatial transcriptomics in *Phragmites australis* reveal the association of B chromosomes with plant invasiveness

**DOI:** 10.1186/s13059-026-04079-x

**Published:** 2026-04-22

**Authors:** Cui Wang, James Ord, Mengxiao Yan, Yunfei Cai, Hongjin Shao, Lele Lin, Jarkko Salojärvi, Lele Liu, Weihua Guo

**Affiliations:** 1 Qingdao Key Laboratory of Ecological Protection and Restoration, School of Life Sciences, Shandong University, 72 Binhai Road, Qingdao, 266237 China; 2 Organismal and Evolutionary Biology Research Program, Faculty of Biological and Environmental Sciences, University of Helsinki, Viikinkaari 1, Biocentre 3, 00790 Helsinki, Finland; 3 Shanghai Key Laboratory of Plant Functional Genomics and Resources, Shanghai Chenshan Botanical Garden, Shanghai, 201602 China; 4 School of Life Science, Qilu Normal University, Jinan, 250100 China; 5 Ecology and Nature Conservation Institute, Chinese Academy of Forestry, No.1 Dongxiaofu, Xiangshan Road, Haidian District, Beijing, 100091 China; 6 School of Biological Sciences, Nanyang Technological University, Singapore, Singapore

**Keywords:** Single cell transcriptomics, Spatial transcriptomics, Invasive plants, B chromosome

## Abstract

**Background:**

Invasive plants pose a major threat to global biodiversity, yet the molecular and genomic mechanisms underlying their success remain poorly understood. Here, we investigate the invasion of the common reed *Phragmites australis*, a grass species that became invasive in North America after its introduction from Europe, to elucidate the molecular mechanisms potentially underlying its invasion process.

**Results:**

By integrating single-cell RNA sequencing, spatial transcriptomics, and comparative genomics, we construct a single-cell atlas of *Phragmites australis*, and identify 19 transcriptionally distinct cell types, including meristematic cells, parenchyma, mesophyll, epidermal, and vascular tissues. Comparative analysis of common garden-grown native populations from Europe and invasive populations from North America reveals a significant proportion of differentially expressed genes located on B chromosomes, which show copy number expansion in invasive genomes. Four B-chromosome genes, *PaChr24B.43* (*IMP-α3*), *PaChr24B.82* (*GATA2*), *PaChr24B.218* (*SCC3*), and *PaChr24B.240* (*SCC3*), are consistently differentially expressed between invasive and noninvasive populations across nearly all cell types. At the tissue level, mesophyll, parenchyma, vascular, and epidermal cells in the invasive population all exhibit increased gene expression in response to sugar starvation and light deprivation. Epidermal cells also show strong activated gene expression in response to hypoxia but suppressed expression of genes involved in the defence related respiratory burst.

**Conclusions:**

This study establishes a cell type-resolved molecular atlas of a nonmodel invasive plant and reveals cell type-specific gene regulatory mechanisms within the regenerative shoot system. These findings advance our understanding of the cellular and genomic basis of plant invasiveness and provide a foundation for future ecological research and management.

**Supplementary Information:**

The online version contains supplementary material available at 10.1186/s13059-026-04079-x.

## Background

Biological invasions pose a profound threat to global ecosystems, as non-native species can disrupt ecological equilibrium, outcompete indigenous flora and fauna, and reshape habitat structures [1]. While many species become invasive upon introduction to new environments, their counterparts may remain non-invasive in their native habitats, as observed in *Spartina* spp., *Ambrosia artemisiifolia*, and *Phragmites australis* [2, 3]. Invasive populations often exhibit distinct phenotypic or reproductive traits in the region in which they have invaded. These contrasting traits, such as altered growth rates, reproductive strategies, or resource use, can play a pivotal role in invasive success [4, 5]. Genetic mechanisms driving such divergence include copy number variation, epistasis, hybridization, and large-effect loci, as well as rapid genomic shifts involving transposable elements and pleiotropy [6, 7]. Structural variants (SVs), including large haplotypic block inversions, are key drivers of rapid local adaptation in invasive species such as *A. artemisiifolia* [8] and *Helianthus* [9]. These adaptive SVs can spread to other populations through hybridization, thereby increasing their potential for survival and range expansion [10]. Understanding the genetic mechanisms underlying the increased invasiveness is therefore crucial for explaining how invasive species establish and outcompete native species in novel environments [11].

The common reed (*P. australis*) is one of the most widespread plant species worldwide and thrives in a variety of wetland habitats across temperate regions. Despite its native status in Europe, where it plays an essential role in wetland ecosystems without exhibiting invasive tendencies, *P. australis* has become a highly aggressive invasive species in North America. Its introduction to the continent is believed to have occurred approximately 200 years ago [3]. Since then, *P. australis* has expanded rapidly across North American wetlands, where it outcompetes native vegetation, reduces biodiversity, and alters hydrological and nutrient cycling processes [12]. One of the key differences observed between invasive and native populations of *P. australis* lies in their regenerative capabilities. In regeneration experiments, invasive populations presented a significantly higher culm regeneration rate than their source populations from Europe [13]. In some cases, invasive populations also exhibit greater rhizome regeneration, further enhancing their ability to colonize new areas and recover from environmental disturbances [14].

Common reeds are characterized by remarkable intraspecific genetic diversity, with substantial variation across genomic loci, ploidy levels, and morphological traits [15–17]. High intraspecific variation in ploidy levels has been detected in common reed, with allotetraploids (2n = 48) and octoploids being the most common. Tetraploids are distributed primarily across Europe, North America, the Mediterranean region, and northern China, whereas octoploids are found mainly in the Far East and southern China. The allotetraploid genomes originated through hybridization between two ancestral diploid species, producing a haploid genome of 24 chromosomes accompanied by a supernumerary B chromosome. Although all contemporary populations are allotetraploids, flow cytometry analyses have revealed that North American *P. australis* populations generally possess slightly larger genomes than their European counterparts [18, 19]. While the exact reasons for the increased genome sizes remain unclear, increasing evidence suggests that an accumulation of B chromosomes and a large number of tandem duplications may play a central role [20].

B chromosomes are supernumerary chromosomes that occur alongside the standard set of A chromosomes. They are typically not essential for survival and often regarded as parasitic [21]. They are known to carry large amounts of repetitive elements and noncoding ribosomal DNA (rDNA). Although B chromosomes are traditionally thought to be genetically inert, recent studies suggest that they may actively participate in gene regulation, potentially causing deleterious effects [22]. B chromosomes can influence the meiosis of plant species by altering the chiasma frequency, recombination rate, and nondisjunction [23]. In maize, the accumulation of B chromosomes has been reported to epigenetically influence A-chromosome gene expression in a tissue-specific manner. These effects can result in white longitudinal striping and reduced fertility, with young leaf tissues showing particular sensitivity to increased B-chromosome copy number [24]. However, the functional role of B chromosomes in invasive species and the mechanisms by which they interact with the broader genome are still poorly understood.

In this study, we aimed to examine whether bioinvasion has altered the gene regulatory networks underlying the regenerative shoot system. To address this, we sampled newly formed shoot regenerated from rhizomes and performed single-cell and spatial transcriptomic analyses on six samples. These included three allotetraploid individuals from the European source population and three from the North American invasive population grown in a common garden. Firstly, by comparing cell-type specific gene expression between invasive and native European populations, we identified differentially expressed genes associated with key developmental processes and different tissue types. This analysis highlights genetic elements in growth and defence pathways that may have been altered following introduction to the new environment. Secondly, we aimed to determine whether B chromosome copy number variation is associated with invasiveness. Thirdly, we examined whether genes on these B chromosomes show evidence of functional divergence from their A- chromosome homologues. For this purpose, we performed comparative genomics, phylogenetics, and selective sweep analyses to investigate the evolution of invasion-related genes via whole-genome and bulk RNA-seq data collected from the same individuals. Our goal was to identify key genes involved in rhizome regeneration and their regulation by B chromosomes, thereby elucidating the genetic basis of *P. australis* invasion. On the basis of the subgenome phasing results from Wang et al. [25], we revised the chromosome and gene annotations to reflect their respective subgenomes, using the suffix A for subgenome A, D for subgenome D, and B for B chromosomes (e.g., *PaChr1A*).

## Results

### Single-cell RNA-seq data for common reed

**Fig. 1:**
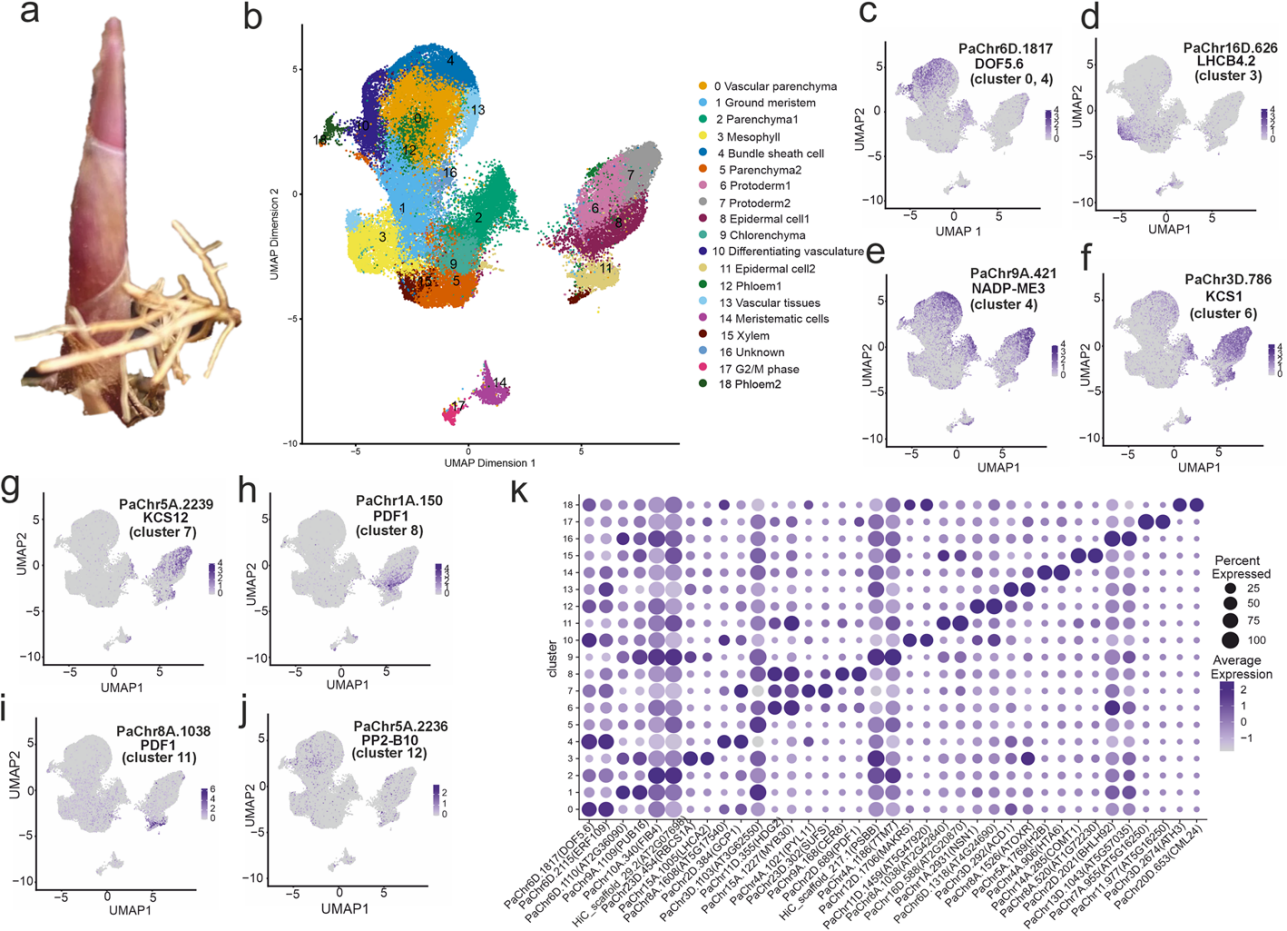
**a** Tissues from common reed shoots were collected for single-cell and spatial transcriptomic analyses. **b** Uniform manifold approximation and projection (UMAP) plot showing the dimensionality reduction of six single-cell samples following batch correction via the Harmony algorithm. The projection is based on 35 principal components (PCs) derived from highly variable genes. Nineteen distinct clusters are depicted in different colours. Panels **c-j** Expression levels of representative marker genes for clusters 0, 3, 4, 6, 7, 8, 11, and 12. **k** Bubble plot displaying the top two most reliable marker genes per cluster. Markers were identified via the FindAllMarkers function in Seurat, using the ROC test to evaluate classification power. The markers were ranked according to the descending ROC score, followed by the average log_2_-fold change (log_2_FC) and cluster-specific expression frequency. Darker blue indicates higher log_2_FC values, whereas bubble size corresponds to relative expression levels within each cluster

The scRNA-seq experiment yielded a comprehensive single-cell atlas of the shoot system of common reeds (Additional file 1: Fig. S1, Fig. 1a). In total, 67,194 cells were captured across the six samples, with per-sample cell counts ranging from 7,154 to 15,297. The EU620 sample presented the highest median gene count, with 3,614 genes per cell, whereas the NAint61 sample presented a median of 2,690 genes per cell (Table 1, Additional file 1: Fig. S2). After removing doublets and cells exhibiting high levels of mitochondrial and chloroplast expression, a total of 57,217 cells were retained for further analysis (Additional file 1: Fig. S3).

**Table 1:** Sample information and sequencing quality for the single-cell transcriptomic dataset

Sample	Group	Data type	Reads mapped to the genome (%)	Reads mapped to the transcriptome (%)	Cell counts	Removed doublets (%)	Median gene per cell	Mean reads per cell
EU60	EU	Single cell	86.1	63.6	11,705	4	3142	30,786
EU78	EU	Single cell	87.4	65.5	12,225	4	3210	29,401
EU620	EU	Single cell	75.9	54.5	7154	6	3614	59,994
NAint61	Invasive	Single cell	83.7	61.4	8645	14	2690	42,478
NAint113	Invasive	Single cell	86.6	62.3	15,297	11	2461	24,167
NAint191	Invasive	Single cell	76.4	54.9	12,168	25	3236	33,900
NAint61	Invasive	Spatial (Subspot level 7)	95.12	88.07	10,867		1736	-

### Single-cell clustering

**Fig. 2:**
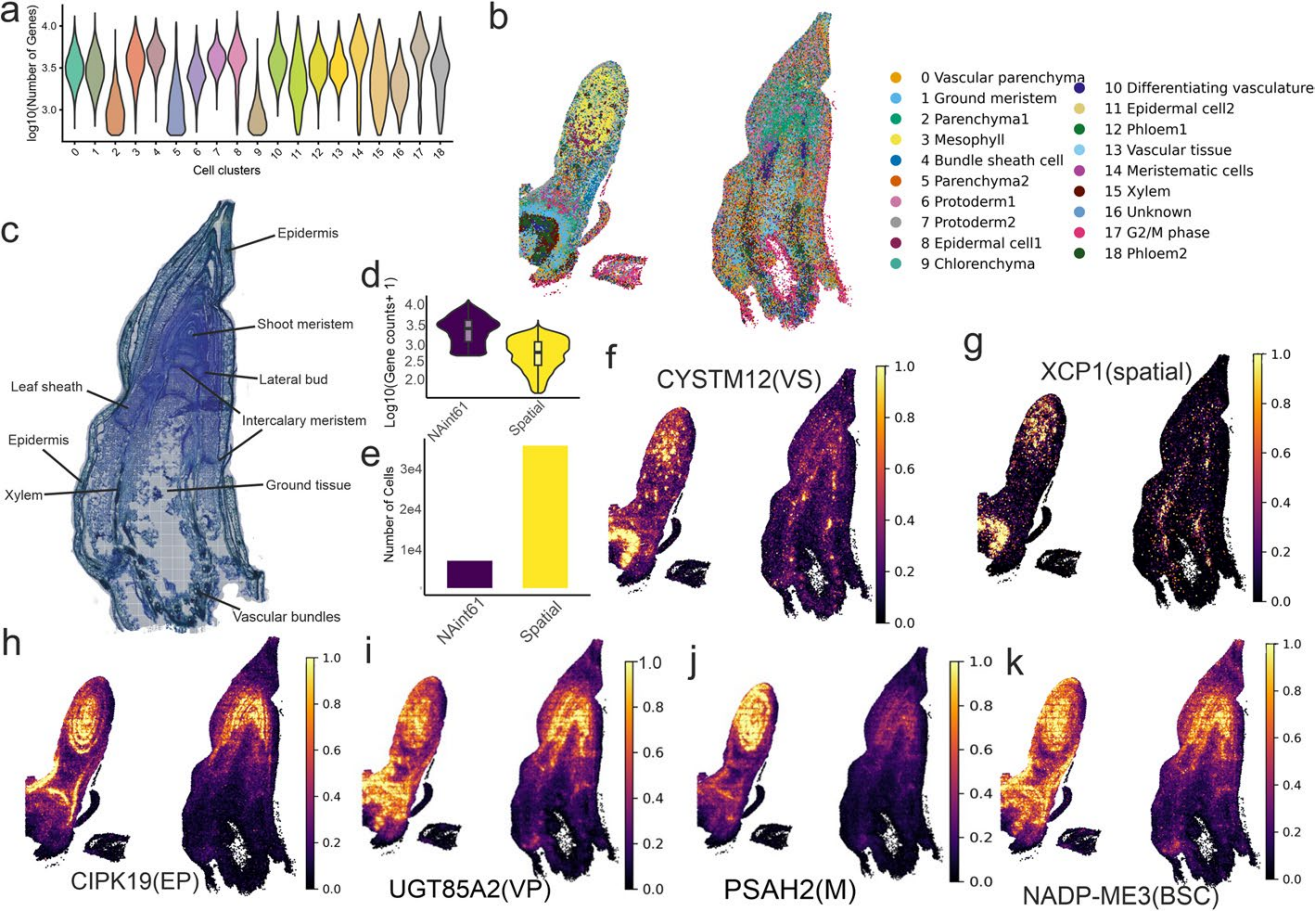
**a** Violin plot showing the distribution of the number of detected genes (log₁₀-transformed) across individual cell clusters. **b** Mapping of single-cell clusters (from NAint61) onto spatial transcriptomic data (sample NAint61) via the Tangram algorithm. The colour legend indicates the corresponding single-cell clusters, with spatial localization reflecting tissue-type associations. **c** Anatomical structure of the shoot from spatial sample NAint61, including the epidermis, shoot apical meristem, lateral bud, intercalary meristem, leaf sheath, xylem, vascular bundles, and undifferentiated ground tissue. **d** Comparison of gene detection distributions between single-cell RNA sequencing and spatial transcriptomics (matrix generated via the 20 μm) in sample NAint61. **e** Comparison of cell number distributions between single-cell RNA sequencing and spatial transcriptomics in sample NAint61. **f-k** Spatial expression patterns of marker genes corresponding to single-cell clusters, projected onto the spatial transcriptomic section. Abbreviations: VS, vascular tissue; EP, epidermal cells; VP, vascular parenchyma; M, mesophyll; BSC, bundle sheath cells

Using a resolution of 0.8, we identified 19 transcriptionally distinct cell clusters. They were visualized through uniform manifold approximation and projection (UMAP) and t-distributed stochastic neighbour embedding (t-SNE) plots (Fig. 1b, Additional file 1: Fig. S4). Cluster-specific marker genes were visualized via UMAP (Fig. 1c–j) and a heatmap (Fig. 1k), and are listed in Additional file 2: Table S1. The cluster-specific cell counts across the six *P. australis* samples from the European (EU) and North American invasive (NAint) populations are summarized in Additional file 2: Table S2. The distribution of individual samples on UMAP is shown in Additional file 1: Fig. S5, and the UMAP distribution of each defined cluster is plotted in Additional file 1: Fig. S6. Overall, cluster 0 consistently presented the greatest number of cells in both the EU and invasive samples. Upon aggregating data from all six samples, we observed that clusters 2, 5, and 9 presented the lowest number of genes expressed per cell, indicating relatively low transcriptional activity. In contrast, clusters 14 and 17 demonstrated the highest gene expression levels per cell, suggesting increased cellular activity (Fig. 2a).

### Spatial transcriptome of shoot tissue

Buds from the NAint61 individual were sectioned for spatial transcriptomic analysis (Fig. 2b, c). Anatomical sectioning of the large bud revealed eight distinct cell types along the vertical plane, including the epidermis, shoot apical meristem, lateral bud, intercalary meristem, ground tissue, vascular bundles, leaf sheath, and differentiating vasculature (Fig. 2c; Additional file 1: Fig. S7). The bud on the left side of the section offered a different perspective of the shoot, with its apical region positioned closer to the transection, enabling the identification of young leaf primordia and associated vascular tissues. We conducted a multiresolution analysis by aggregating different numbers of spatial barcoding spots into nine levels of subspots, each serving as an analytical unit. Summary statistics, including the number of subspots, median Unique Molecular Identifiers (UMI) counts per subspot, median gene counts per subspot, and total number of detected genes, are presented in Additional file 2: Table S3. In comparison, the single-cell RNA-seq dataset yielded a median gene count per cell ranging from 2,461 to 3,614 (Fig. 2a). To integrate spatial resolution with cellular identity, we generated a spatial subspot dataset with a spot diameter of 20 µm. This approach identified 37,324 cells, with a median of 561 genes per cell and a median UMI count of 655. Although the number of genes per cell was lower than that in the single-cell dataset, this method enabled the capture of a substantially larger number of cells (Fig. 2d, e). The highest gene expression levels were detected in young, actively growing tissues, such as meristematic regions and protoderms (Additional file 1: Fig. S7). Spatial transcriptomics analysis revealed 17 distinct spatial clusters corresponding to the anatomical structure (Additional file 1: Fig. S8). The marker genes for each spatial cluster were identified at a 20 µm resolution and are summarized in Additional file 2: Table S4.

### Cell type identification from single-cell data and spatial projection

We annotated the cell types of each single-cell cluster on the basis of molecular markers reported in literature (Table 2; Additional file 2: Table S1). To further validate spatial identity, representative marker genes from each single-cell cluster were projected onto the spatial transcriptomic data, as shown in Fig. 2f–k. To map transcriptional profiles onto anatomical structures, gene expression signatures from the single-cell RNA-seq clusters were projected onto the spatial transcriptomic dataset (Fig. 2b; Additional file 1: Fig. S9). Eight rounds of projection were performed to optimize the mapping. The best mapping performance (score = 0.825) was achieved via single-cell data from the donor NAint61, which were mapped to a 20 μm spatial resolution. These single-cell and spatial transcriptomics data were derived from the same individual, and the model was trained on 1,000 highly variable genes (Additional file 1: Fig. S10, Additional file 2: Table S5). The Tangram cluster-to-space mode provided higher-resolution spatial projections than the cell-to-space mode, despite its lower quantitative mapping score (Additional file 1: Fig. S11). Marker genes showed consistent Pearson and Spearman correlations between the predicted and measured spatial gene expression, with markers from clusters 3, 8, 9, 11, and 18 showing the highest Pearson correlation and mapping scores. (Additional file 2: Table S6).

**Table 2:** Classical markers for each cell type

Cluster	Cell type	Gene markers	Reference
0	Vascular parenchyma	*DOF zinc finger protein* (*DOF5.6*)	[26, 27]
1	Ground meristem		
2	Parenchyma1		
3	Mesophyll	*ribulose bisphosphate carboxylase small chain 1A* (*RBCS1A*), *chlorophyll a-b binding protein CP29.2* (*LHCB4.2*), *chlorophyll a-b binding protein CP26* (*LHCB5*), *light-harvesting chlorophyll B-binding 2* (*LHCB2*), *light-harvesting chlorophyll B-binding protein 3* (*LHCB3*), *photosystem I chlorophyll A/B-binding protein 1* (*LHCA2*), *photosystem I reaction centre subunit III* (*PSAF*), and *photosystem I subunit E-2* (*PSAE-2*)	[27, 28]
4	Bundle sheath cells	*NADP-dependent malic enzyme 3* (*NADP-ME3*), *scr-like 23* (*SCL23*), *phenylalanine ammonia-lyase1* (*PAL1*)	[27, 29–35]
5	Parenchyma2		
6	Protoderm1	*KCS1*, *KCS11*, *KCS12*, *FDH* (*KCS10*), *homeodomain glabrous 2* (*HDG2*)	[27, 35–38]
7	Protoderm2	*KCS1*, *KCS11*, *KCS12*, *FDH* (*KCS10*),* AtLTP1, LACS4*	[27, 28, 36–40]
8	Epidermal cell1	*KCS1*, *KCS11*, *KCS12*, *FDH* (*KCS10*)**,** *Arabidopsis protodermal factor 1* (*PDF1*), *hydroxysteroid dehydrogenase 1* (*HSD1*)	[27, 28, 35–38, 41]
9	Chlorenchyma		
10	Differentiating vasculature	*ATHB-5*	[28, 42]
11	Epidermal cell2	*KCS1*, *KCS11*, *KCS12*, *FDH* (*KCS10*), *Arabidopsis protodermal factor 1* (*PDF1*), *long-chain acyl-CoA synthetase 2* (*LACS2*)	[27, 28, 35–38, 41]
12	Phloem1	*phloem-specific marker PHLOEM PROTEIN 2-B10* (*PP2-B10*)	[43]
13	Vascular tissue	*ATHB-7*	[28, 42]
14	Meristematic cells	*high mobility group B protein 1* (*AtHMGB1*)	[44]
15	Xylem	*UDP-xylose synthase 6* (*UXS6*), *AtCOMT*	[45]
16	Unknown		
17	G2/M phase	*cyclin-dependent kinase* (*CDKB*)	[46]
18	Phloem2	*DOF5.3*	[47]

To gain further insight into cluster identity, we performed Gene Ontology (GO) enrichment analysis on the basis of cell markers and analysed the spatial locations of each single-cell cluster (Additional file 1: Fig. S12). For cluster 1, enriched GO terms were related to responses to oxygen-containing compounds, abiotic stimuli, lipids, and hormones. Spatial projection placed these cells in the ground tissues between intercalary meristems and in the pith region, suggesting that they were ground meristems. Clusters 2 and 5 are located beneath cluster 1 in the leaf sheaths, ground tissue, and epidermis in the spatial projection, with only one to five markers discovered. While it is difficult to precisely identify the exact cell types, their spatial localization on the anatomical structure suggests that they likely represent parenchyma tissues. Cluster 9 had few markers but projected to mesophyll regions, and trajectory analysis supported its identity as developing chlorenchyma. Cluster 10 was mapped to differentiating vascular tissue in the spatial data and exhibited the expression of vascular marker genes (Table 2). The marker genes of cluster 14 were enriched for biological processes such as translation, negative regulation of DNA recombination, nucleosome assembly, macromolecule biosynthesis, negative regulation of DNA metabolic processes, protein-DNA complex assembly, chromatin organization, heterochromatin organization, organonitrogen compound biosynthesis, cell division, cellular biosynthetic processes, DNA dealkylation, DNA demethylation, and RNA metabolism, suggesting an active role in cell proliferation. Its spatial projection to the shoot meristem supported its annotation as a meristematic cell. Cluster 15 exhibited GO enrichment in the S-adenosylmethionine metabolic process and was projected to the vascular system. Given the known role of S-adenosylmethionine in lignin biosynthesis, which is characteristic of lignified tissues, we classified this cluster as xylem [48], which was further verified by spatial projection. Cluster 16, enriched for genes related to responses to external biotic stimuli, remains unclassified. Cluster 17 was enriched for GO terms related to translation, organonitrogen compound biosynthetic processes, cytoplasmic translation, ribonucleoprotein complex biogenesis, cellular component organization or biogenesis, regulation of the cell cycle, mitotic phase transition, translational elongation, cellular component assembly, regulation of DNA-templated DNA replication, and cytoskeleton organization. These functions suggest that cluster 17 is a cell cycle active population with high proliferative potential. Spatially, it projected to the lateral bud meristem. Cluster 18, which was projected to vascular tissues and exhibited high similarity to companion cells in *Arabidopsis*, was confirmed as phloem cells.

### Developmental pathways

For clarity and consistency, all clusters discussed in this article refer to single-cell data unless explicitly described otherwise. RNA velocity analysis, derived from the ratio of spliced to unspliced transcripts, was used to infer dynamic cell state transitions. Using the meristematic cells (cluster 14) as the initial cluster, clusters 18 and 11 consistently emerged as terminal-like states in 50% and 33% of the samples in the cell lineage trajectories, respectively. Given that developmental programs are typically conserved within a species, we selected EU620 as a representative sample to explore cell type specific gene drivers. Cell fate mapping via CellRank identified three clusters with high fate probabilities (Additional file 1: Fig. S13), prominently including lineage 1 (mostly clusters 1, ground meristem), lineage 11 (mostly cluster 11, epidermal cells), and three subpopulations within lineage 9 (mostly cluster 9, chlorenchyma). The identification of three distinct subgroups within cluster 9 suggests cellular heterogeneity within this cluster.

**Fig. 3:**
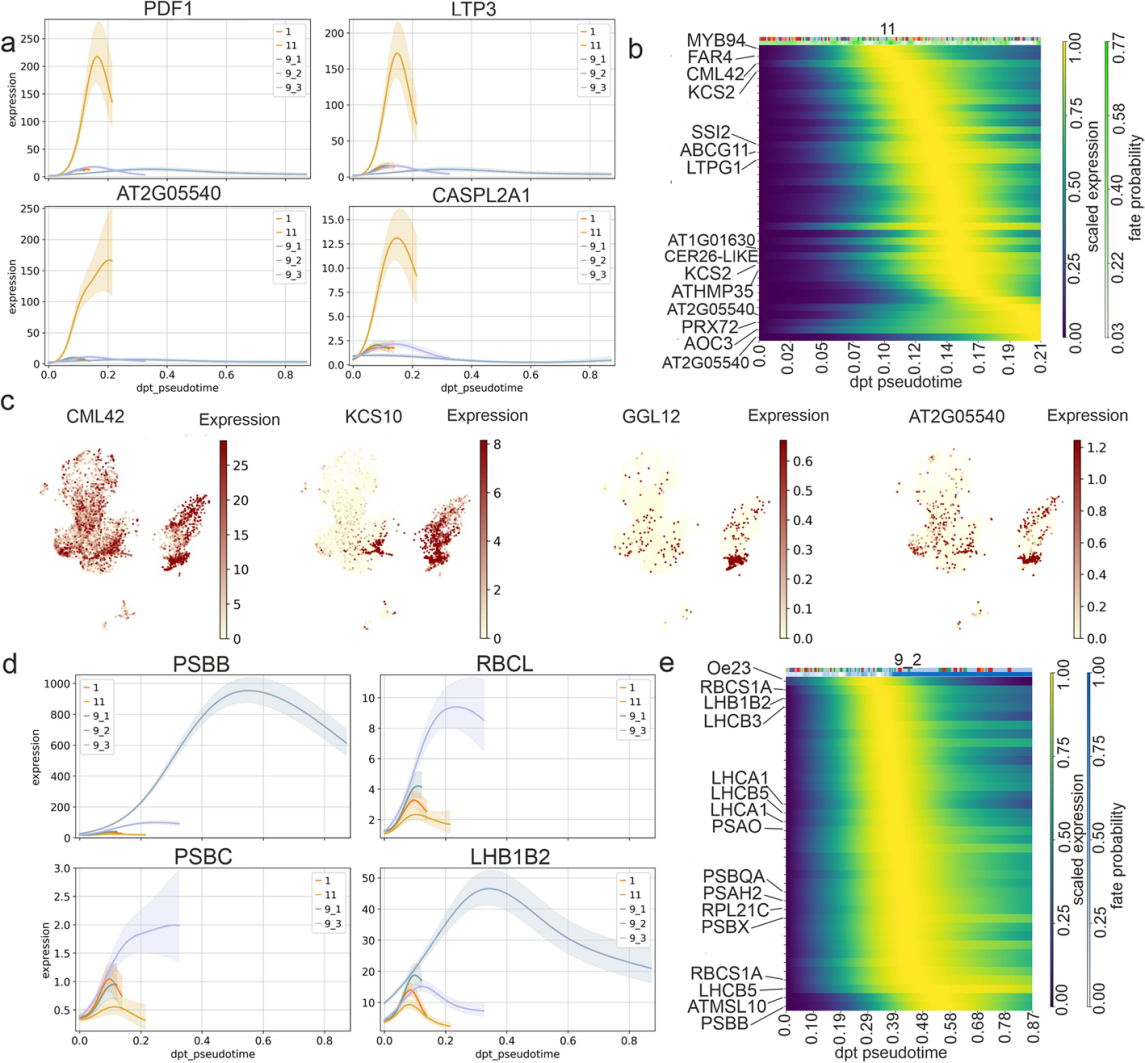
**a** Dynamic expression patterns of key driver genes in lineage 11 across its developmental trajectory, including *PaChr8A.1038* (*PDF1*), *Chr10.1228* (*LTP3*), *PaChr6D.1298* (*AT2G05540*), and *PaChr11D.1614* (*CASPL2A1*). **b** Representative gene expression profiles along the pseudotime trajectory, highlighting cell fate determination genes using lineage 11 as the terminal state. The yellow shaded region indicates the time window of peak gene expression. **c** UMAP plots showing the spatial expression of sequentially activated cell determination genes during the development of lineage 11: *PaChr3D.1219* (*CML42*), *PaChr3D.474* (*KCS10*), *PaChr13D.837* (*GGL12*), and *PaChr6D.1299* (*AT2G05540*). **d** The top driver genes in lineage 9 exhibit peak expression at distinct stages along its pseudotime developmental trajectory. **e** Temporal expression dynamics of genes associated with cell fate determination in lineage 9 (cell state 2) along the pseudotime trajectory. The yellow highlight indicates the point of peak expression, whereas the dark blue shading represents the probability of the gene’s involvement in fate determination

In CellRank, a lineage-specific driver gene is a gene whose expression is significantly correlated with the fate probability along a particular lineage, indicating its association with or potential influence on differentiation toward that cell fate [49]. The key driver genes associated with the development of lineage 11 included *PDF1* (*protodermal factor 1*), *LTP3* (*lipid transfer protein 3*), and *CASPL2A1* (*Casp-like protein 2A1*), whose expression peaks at the onset of the differentiation, and a homolog of *AT2G05540* (*glycine-rich protein family*), whose expression peaks at the turning point (Fig. 3a). Pseudotime analysis of lineage 11 revealed progressive upregulation of lipid biosynthesis regulators (*MYB94*, *FAR4*, and *KCS2*) to produce cuticular wax, initiators of trichome branching (*CML42*), adjustment of lipid compositions in the epidermis (*SSI2*, *ABCG11*, and *LTPG1*), metal ion transportation (*CER-26-LIKE*, and *ATHMP35*), and activation of vascular system fate genes (*PRX72*) as well as plant structural development regulators (*AOC3*) (Fig. 3b). The expression of the diver genes also progressively increased from multiple cell clusters to lineage 11 (Fig. 3c). The top drivers of lineage 9 included homologs of *MT2B*, *ATHCYSTM12*, *NRX1*, and *AT3G17020* at cell state 1; *PSBB*, *RBCL*, *PSBC* and *LHB1B2* at cell state 2; and *FIB4*, *DGK1*, *nPAP* and *LRK10L-2.8* at cell state 3 (Additional file 1: Fig. S14). In lineage 9, cell state 2 demonstrated the expression of numerous genes encoding photosystem components, which are well-established mesophyll markers. These genes also presented high probabilities of cell fate determination (Fig. 3d, e), indicating a strong commitment to mesophyll identity. Given the pronounced heterogeneity in photosystem-related gene expression within cluster 9, we propose that this cluster represents chlorenchyma tissue in *P. australis*.

### Differentially expressed genes between the EU and invasive lineages

**Fig. 4:**
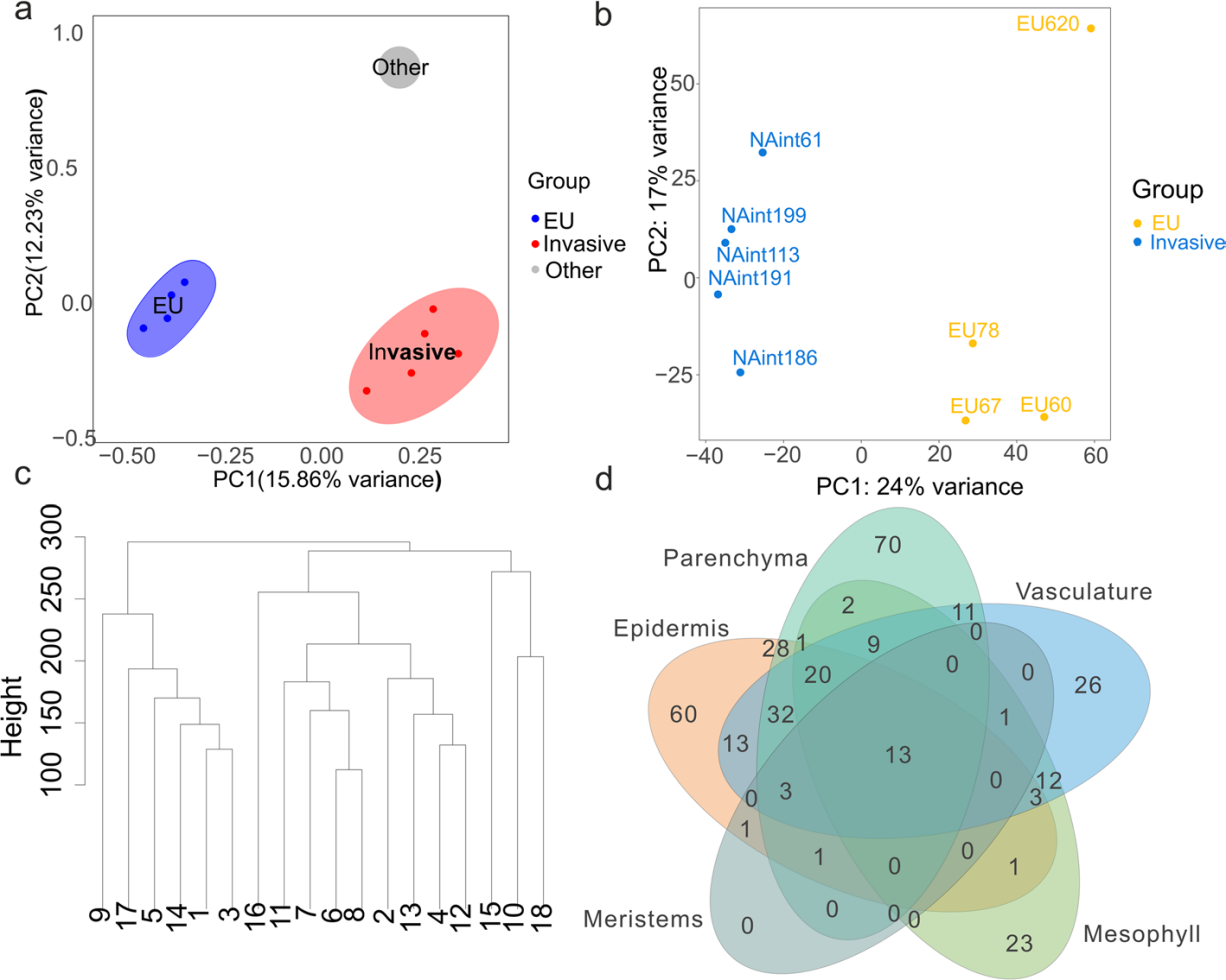
**a** Principal component analysis (PCA) plot based on SNP variants obtained by aligning short reads from whole-genome sequencing to the reference genome. The samples enclosed in the blue ellipse represent European (EU) populations, whereas those in the red ellipse represent invasive populations. An additional sample, not enclosed, corresponds to a whole-genome sequence from an invasive individual reported in Oh et al. [52]. **b** PCA plot based on gene expression levels from leaf tissues mapped to the reference genome. A significant portion of the variance is explained by the separation between population groups, although gene expression variability is also observed within the EU group. **c** Dendrogram depicting the relatedness of single-cell clusters, constructed via the hclust function with the complete linkage method. **d** Venn diagram showing genes upregulated in the invasive population across five tissue types across six samples: vascular tissue, parenchyma, epidermis, meristematic cells, and mesophyll. The vascular tissues included clusters 4, 10, 12, 13, 15, and 18; the epidermal tissue included clusters 6, 7, 8, and 11; the parenchyma included clusters 2, 5, 9, and 16; the meristems corresponded to cluster 14; and the mesophyll corresponded to cluster 3. Genes with an adjusted *p* value (FDR) < 0.05 and |log₂-fold change|> 2 were considered significantly differentially expressed

In the comparison of single-cell transcriptomic data between European and North American invasive populations, the invasive lineages generally presented a slightly lower median number of cells per sample (Additional file 2: Table S2). To ensure robust differential expression analysis, we aggregated single-cell RNA counts within each cluster to generate pseudobulk counts per sample. Compared with conventional single-cell models, this method reduces the number of multiple comparisons across thousands of cells, thereby minimizing false positives and improving accuracy [50]. Principal component analysis (PCA) of the pseudobulk expression profiles distinguished invasive and non-invasive lineages, but a substantial proportion of variation was still observed within populations (Additional file 1: Figs. S15, S16). This pattern deviates slightly from the PCA results based on whole-genome sequencing (WGS) variants and leaf bulk RNA-seq, in which the largest proportion of variation was observed between groups (Fig. 4a, b), indicating possible batch effects or biological heterogeneity within groups. In support of this interpretation, RNA velocity analysis revealed that sample EU620 displayed distinct transcriptional dynamics compared with other European samples (Additional file 1: Fig. S17). The gene expression profiles of non-invasive EU620 samples clustered more closely with those of the two invasive samples, raising the possibility of artifacts such as differences in sequencing depth or coverage.

Using correlations in gene expression among closely related cell types, we constructed a dendrogram to visualize their relationships on the basis of a gene-by-cell-type matrix of average relative abundance (Fig. 4c). The topology may vary with different individuals, but clusters 6, 7, 8 and 11, as well as clusters 1 and 3, are stably grouped in the same clusters. We assigned each cluster to broader tissue categories for differential gene expression analysis: parenchyma (clusters 2, 5, 9, 16), epidermis (clusters 6, 7, 8, 11), vascular tissue (clusters 4, 10, 12, 13, 15, 18), meristem (cluster 14) and mesophyll (cluster 3).

The mean number of reads per cell was calculated as the total number of sequenced reads divided by the number of cells in a sample. Since EU620 had a greater mean read depth per cell but lower cell counts, whereas NAin113 showed the opposite trend, we included the mean reads per cell as a covariate in the differential expression analysis. The inclusion of sequencing depth as a covariate improves DESeq2 differential expression analysis by mitigating batch effects from varying sequencing coverage, as shown by methodological benchmarking [51]. Genes with a |log₂FC|> 2 and an FDR-corrected *p*-value < 0.05 were classified as DEGs for each cluster (Additional file 2: Table S7). We also evaluated alternative parameter settings and analyses with outliers removed, which yielded a high overlap among the recovered DEGs (Additional file 2: Table S8-S10). For superclusters, a more relaxed threshold of |log₂FC|> 1 was applied. The parenchyma presented the greatest number of DEGs, with 190 upregulated and 80 downregulated DEGs in the invasive population (Fig. 4d; Additional file 2: Tables S11-S14). The genes upregulated in the invasive lineages were enriched for response to the absence of light, sucrose starvation, and plant-type cell wall organization. Specifically, 30 (15.8%) of these genes were located on the B chromosome. In the epidermis, 176 genes were upregulated, with 25 (14.2%) being located on the B chromosome, and 89 were downregulated. The upregulated genes were associated with the hypoxia response, sucrose starvation, and absence of light, whereas downregulated genes were linked to defence responses against bacteria (Additional file 2: Table S13). Vascular tissues presented 143 upregulated genes (29 of which [20.2%] were on the B chromosome) enriched for similar pathways, including sucrose starvation and the absence of light. Among the cluster 3 mesophyll cells, 85 genes were upregulated (18 [21.2%] on the B chromosome), and 25 were downregulated in the invasive population.

At a finer cluster resolution, upregulated genes in invasive samples were predominantly involved in sucrose starvation response (clusters 3, 11), spindle attachment and meiotic cohesion (clusters 7, 9, 10, 12, 14, 16, 17), responses to oxygen-containing compounds, fatty acids, and jasmonic acid (clusters 8, 11), brassinosteroid/gibberellin signalling and olefinic compound metabolism (cluster 8), as well as L-asparagine metabolism (cluster 11). The downregulated genes were not significantly enriched for any GO terms in most clusters, except for cluster 8, where the downregulated genes were enriched for "respiratory burst involved in defence response", and cluster 11 that exhibited broad functional enrichments, encompassing three major categories: (1) stress and defence responses, including defence responses to bacteria, fungi, and other organisms, as well as responses to oxidative stress, osmotic stress, and water deprivation; (2) responses to hormonal and chemical stimuli, such as abscisic acid, alcohol, oxygen-containing compounds, and lipids; and (3) regulation of biological processes, including defence and stress response pathways (Additional file 2: Table S15).

### DEG analysis of mature leaves between the two groups

Bulk RNA-seq data obtained from mature leaf tissue of the same individuals clearly separated the European and North American populations on the first axis of the PCA (Fig. 4b). We found that 147 genes were significantly upregulated in the invasive lineage, among which 68 (46%) mapped to the B chromosome (Additional file 2: Tables S16, S17), and four genes were enriched for the biological process "fusion of sperm to egg plasma membrane involved in double fertilization forming a zygote and endosperm". Ninety-seven genes were downregulated in the invasive population, with significant enrichment for multiple biosynthetic pathways, including isoprenoid, hydrocarbon, terpenoid, sesquiterpenoid, and diterpenoid biosynthesis, as well as biological processes such as the response to herbivores and the defence response to other organisms.

### B chromosome copy number variation and gene expression profiles

**Fig. 5:**
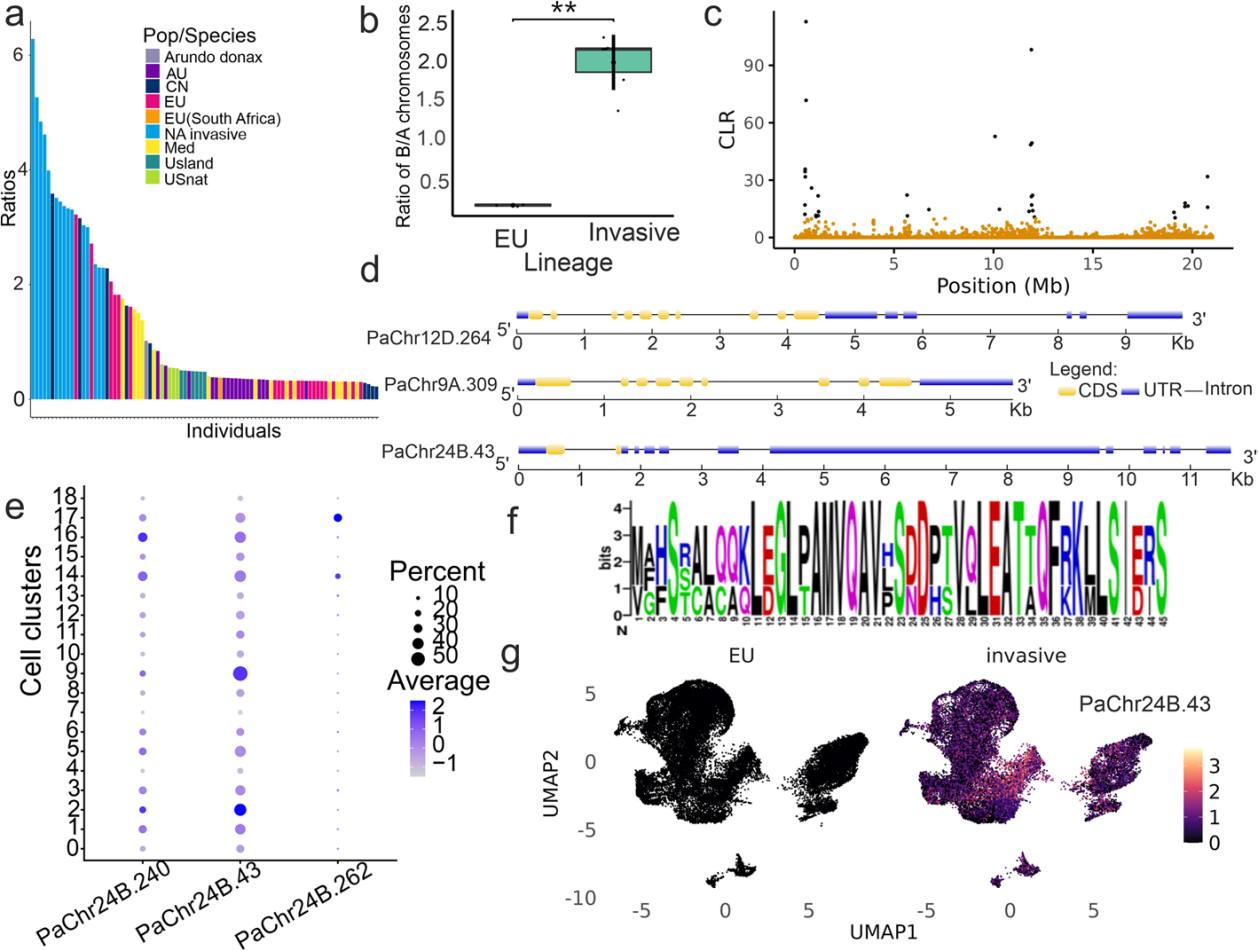
**a** Bar plot showing the copy number of B chromosomes across different genetic lineages of *P. australis*. The blue bars represent invasive individuals in North America, all of which presented higher B chromosome copy numbers than the other groups. **b** Comparison of the ratio of reads mapped to B chromosomes versus those mapped to A chromosomes between invasive and non-invasive European populations. Statistical significance was assessed via the Mann–Whitney U test. **c** Genome scan for selective sweeps on the B chromosome. SweepFinder2 uses the genome-wide neutral site frequency spectrum (SFS) as a reference and then evaluates each genomic position by computing the CLR between a neutral model and a selective sweep model. A higher CLR indicates that the local allele-frequency pattern deviates more strongly from the neutral baseline and better matches expectations under a selective sweep. The CLR for a selective sweep was calculated in 5-kb sliding windows across the B chromosome, on the basis of the SFS derived from whole-genome sequencing of an invasive population. Each dot represents the CLR value for a single window. The black dots highlight windows with a CLR value exceeding the significance threshold of 10, indicating putative regions under positive selection. **d** Gene structure of homologues *PaChr12D.264*, *PaChr9A.309*, and *PaChr24B.43*. Schematics showing the organization of coding sequences (CDSs), untranslated regions (UTRs), and introns. *PaChr24B.43* represents a derived locus originating from the homoeologous copies *PaChr12D.264* and *PaChr9A.309*. Arrowheads indicate transcriptional orientation; nucleotide lengths above exons are shown. **e** Bubble plot showing the average expression levels (log₂ fold changes) of three B chromosome genes across clusters. A darker blue colour indicates greater average expression, whereas bubble size reflects the proportion of cells in which the gene is expressed. **f** Conserved amino acid regions of *PaChr24B.43* among homologous genes in *P. australis* and *Zea mays*. **g** Expression of B chromosome genes *PaChr24B.43* projected onto UMAP space. Gene expression levels (dots) are overlaid on the UMAP for non-invasive and invasive individuals, showing elevated expression in cells from invasive individuals

Mapping of RAD-seq reads from 88 globally sampled individuals to the reference genome revealed that invasive individuals consistently had a higher read depth ratio of B chromosomes to autosomes than other populations (Fig. 5a). This observation was further confirmed through whole-genome sequencing of nine individuals, including the ones used for single-cell and bulk transcriptomic analyses (Additional file 2: Table S18). The B chromosome-to-autosome read depth ratio averaged 1.99 ± 0.335 in the invasive lineages, indicating an average of four B chromosome copies. In contrast, the EU population presented a ratio of 0.261 ± 0.0115, suggesting the near absence of B chromosomes (Fig. 5b). We next identified selective sweeps in the invasive population by running SweepFinder2 on the unfolded minor-allele-frequency spectrum. We identified altogether 86 selective sweeps in the invasive population (windows with exceptionally high composite likelihood ratios, CLR > 10), of which 84 overlapped with repeat regions and 25 with genes, respectively, indicating positive selection on these regions (Fig. 5c). Alignment coverage in repeat regions was significantly greater than the genome-wide average (Wilcoxon rank-sum test, *p* < 2.2e^–16^), suggesting either multimapping or possible false positives. Altogether, 34 of the high-CLR windows were located on the B chromosomes, 32 of which overlapped with repeats. Thirteen genes on the B chromosome were identified as directly under selective sweep (CLR > 10), including *PaChr24B.17* (*VFB3*), *PaChr24B.18* (*ATPD*), *PaChr24B.19* (*AT3G49050*), *PaChr24B.52* (*DL4605C*), *PaChr24B.110* (CIP111), *PaChr24B.173* (*GEX2*), *PaChr24B.174* (*At1g77550*), *PaChr24B.177* (*CRK8*), *PaChr24B.183, PaChr24B.188* (*AT5G56050*)*, PaChr24.187* (*GEX3*)*, PaChr24.197* (*OEP80*)*,* and *PaChr24.230* (*AtC3H64*)*.* Genes under selective sweep rarely overlapped with highly expressed genes in the invasive population; all highly expressed B chromosome genes had CLR values below 6, mostly near zero, indicating that they are unlikely to be recent targets of positive selection. In the European source population, 30 windows of high CLR were detected; 29 of which were overlapping repeats, 7 of which also overlapped genes. No windows were shared between the two populations.

Pseudobulk differential expression analysis across the clusters, found 33 out of 286 genes on the B chromosome that are upregulated in the invasive population and function differently in different cell types (Additional file 2: Table S19). The genes *SCC3* (*PaChr24B.240* and *PaChr24B.218*), *IMP-α3* (*PaChr24B.43*), and *GATA2* (*PaChr24B.82*) presented significantly increased expression in the invasive lineage across nearly all clusters. The *OMTF3* gene, which encodes a methyltransferase, and *ABI5*, a transcription factor involved in ABA signalling, were exclusively upregulated in the epidermal cells of cluster 8. The *MER3* gene, encoding a DNA helicase, was specifically upregulated in the meristem (cluster 14).

Phylogenetic analysis of importin genes placed *PaChr24B.43* in a separate clade distinct from its homologs on chromosomes 9 (*PaChr9A.309*) and 12 (*PaChr12D.264*), indicating that *PaChr24B.43* originated from ancestral gene duplication but exhibited an accelerated evolutionary rate, as reflected by its long branch length (Additional file 1: Fig. S18). A detailed examination revealed that the 402 bp coding sequence (CDS) of *PaChr24B.43* aligns with two continuous regions in each of the two homologous genes but has acquired a long insertion between these fragments after duplication from the ancestral copies (Fig. 5d). Alignment of its mRNA sequence with homologues from closely related species (including rice, sorghum, maize, and *Setaria viridis*) indicated that *PaChr24B.43* encodes a truncated protein (Additional file 1: Figs. S19, S20). This truncation results from a late start codon and a premature stop codon caused by a frameshift mutation at an exon intron boundary. Although the translated protein begins with conserved sequences, it is followed by random amino acids until early termination (Fig. 5f). A segment of the protein (corresponding to nucleotides 208–369) displays homology to the *IMP-α3* gene in other species, albeit with low sequence identity (< 60%). Given its partial cross-species homology and truncated structure, we conclude that *PaChr24B.43* is likely a pseudogene.

*PaChr24B.240* and *PaChr24B.218*, both encoding the kinetochore-related SCC3 protein, clustered together with *PaChr10A.167* and *PaChr21D.972*. Similarly, *PaChr24B.262*, which encodes a DDE family endonuclease, formed a clade with *PaChr17A.331*. The phylogenetic relationships of *PaChr24B.218* and *PaChr24B.240* are further illustrated in Additional file 1: Fig. S21. The expression levels of the top three ranked B chromosome genes are summarized and visualized on the UMAP plots in Fig. 5e and g.

## Discussion

By integrating scRNA-seq with spatial transcriptomics, we projected single-cell data onto spatial coordinates with high accuracy, enabling confident cell type annotation. Since the two technologies are intrinsically different and do not correspond exactly, each spatial spot is often treated as equivalent to a single cell for comparison with single-cell data. In our case, the NAint61 single-cell dataset captured far more genes per cell but contained fewer total cells than the spatial data, reflecting the technical differences between scRNA-seq and spatial transcriptomics. ScRNA-seq methods, such as droplet-based methods, isolate and lyse intact cells and recover most cytoplasmic and nuclear mRNAs, whereas spatial transcriptomics profiles RNA directly from tissue sections. Because a 20 µm slice typically intersects only part of a 10–20 µm cell, each spatial spot represents transcripts from partial cellular content rather than a complete cell. Given that our spatial transcriptomic dataset offered multiple resolutions, we assessed which resolution best matched the single-cell data during spatial projection.

Comparing different integration strategies to map scRNAseq clusters onto spatial transcriptomic data, we found that using each sample's own single-cell dataset yielded slightly higher accuracy than using data from closely related individuals, indicating within-sample matching improves mapping precision. We further evaluated the impact of spatial resolution by comparing coarse (37 µm), fine (20 µm), and cell-split datasets. The fine-resolution data consistently achieved the highest mapping scores, demonstrating that greater spatial detail enhances alignment accuracy. Interestingly, the cell-split dataset, which was anticipated to be optimal, demonstrated lower mapping efficiency. This is likely attributable to the heterogeneous sizes of plant cells and the presence of a hollow pith region, which collectively complicate accurate cell segmentation. In this study, cell types annotated through spatial transcriptomics were largely consistent with canonical cell markers. Nevertheless, caution should be exercised when discrepancies arise between cell type identities inferred from spatial and single-cell data.

In this study, we identified multiple cell types within the shoot system. These included vascular tissues, parenchyma, meristematic cells, mesophyll, ground meristem, bundle sheath cells, phloem, xylem, protoderm, and epidermal cells. The developmental trajectories of multiple tissues, inferred from the scRNA-seq dataset provided molecular insights into the differentiation of the chlorenchyma and epidermis. Chlorenchyma (cluster 9) is a type of parenchyma tissue that contains abundant chloroplasts and is typically found in the mesophyll of leaves. It plays a vital role in photosynthesis by capturing light energy and synthesizing organic compounds. The identification of three distinct cell states spanning a broad pseudo-temporal range in this cluster suggests that the chlorenchyma may retain multipotency and the potential to differentiate into multiple lineages [53]. These findings align with those of a study on tomato callus via spatial transcriptomics, which revealed that chlorenchyma cells contribute to complete shoot system development in tomato [54]. In addition to chlorenchyma, bundle sheath cells and vascular parenchyma are considered key sources of regeneration in Poaceae species [55]. In contrast, epidermal cells underwent typical developmental transitions from protoderms (clusters 6 and 7) to more specific differentiated tissues (clusters 8 and 11). Following typical epidermal cell development in plants, the cell fate determination and morphogenesis of epidermal cells are first regulated by the critical regulator *PDF2*, which is a cell marker of the protoderm clusters [56]. The developmental trajectory of mature epidermal cells (cluster 11) involved early gene expression related to trichome branching. This conforms with the notion that trichome differentiation may occur earlier than that of other stomatal cells [56].

Spatial mapping of single-cell cluster 1 to spatial clusters 11, 0, and 3 (ground tissue) indicated that, in addition to its role as a ground meristem, it is also likely associated with aerenchyma formation. In the marsh aquatic plant *Typha angustifolia* L. (Typhaceae), the development of leaf aerenchyma is divided into four stages on the basis of morphological characteristics: solid cavity formation, early cavity formation, late cavity formation, and mature cavity formation [57]. Our data identified *APX1* as a cell-type marker for spatial clusters 11. *Prx52*, a protein similar to peroxidases involved in lignin biosynthesis, was also found within this cluster. These findings suggest that cluster 1 in the single-cell data may initiate aerenchyma formation during the solid stage, as this stage is characterized by high expression levels of *APX* and *Prx* genes, which decrease as development progresses [57]. The anatomical structure also revealed a looser tissue arrangement on the lower side of the shoot, which is consistent with the cavity formation process observed in common reeds [58].

Developmental system drift, defined as the genetic divergence in the developmental pathways of homologous phenotypic traits over evolutionary trajectories [59, 60], was observed in shoot development between the two populations of common reed. This was reflected in differential gene expression across distinct cell types between groups, with a notable involvement of B chromosome genes in the invasive population. Compared with non-invasive European populations, invasive populations of *P. australis* exhibit enhanced tolerance to hypoxic conditions and a downregulation of genes linked to acute defence responses. Additionally, we observed a tendency for elevated expression of genes associated with photosynthetic activity; however, this particular inference is based on only two replicates per group and should therefore be interpreted with caution (Additional file 3: Supplementary Note 1). This pattern aligns with life history trade-off theory, which suggests that energetically costly defensive traits are often lost in favour of traits that support rapid growth and high fecundity. A similar shift has been observed in *Ageratina adenophora*, where invasive populations allocate more nitrogen to photosynthetic activities rather than structural cell construction, unlike their native counterparts [61]. This aligns with the broader finding that invasive species exhibit superior resource efficiency, as demonstrated by a comparative study of 92 species pairs, which revealed that they evolve higher photosynthetic rates and more efficient use of energy, nitrogen, phosphorus, and potassium [62]. For common reed, the invasive population was found to differ from its European ancestor in terms of photosynthetic nitrogen use efficiency and structural investment costs [63].

The invasive population presented upregulated gene expression related to sucrose starvation, hypoxia, and the absence of light in most cell types, coupled with a downregulation of genes involved in the respiratory burst and response to alcohol in epidermal cells. This pattern demonstrates a comprehensive stress tolerance strategy. Under hypoxic conditions, plants switch to anaerobic respiration due to impaired TCA cycle activity, leading to ethanol (alcohol) production. Epidermal cell-downregulated genes enriched in "responses to alcohol" help mitigate ethanol toxicity, reducing cellular damage. This adaptive mechanism allows invasive plant populations to better survive in oxygen-deprived environments by optimizing energy use and protecting against alcohol-induced injury.

The upregulation of B chromosome genes in the invasive population was driven by copy number variation, with a greater number of B chromosomes in the invasive population. These genes are involved in essential processes such as DNA repair, gamete formation, methylation, photomorphogenesis, hormone responses (auxin, brassinosteroids, gibberellic acid), and hypoxia tolerance, creating a hotspot that may facilitate rapid adaptation to new environments. The B chromosome gene *PaChr24B.43* is homologous to *IMPORTIN-α3/MOS6 (MODIFIER OF SNC1, 6)* in *Arabidopsis*, a key player in plant innate immunity that is associated with rapidly evolving resistance genes [64–67]. While its conserved paralogue on Chromosome 9 shows no differential expression, *PaChr24B.43* itself is undergoing rapid evolution. Compared with the homologous *PaChr9A.309* (which contains 531 amino acids), it has only 133 amino acids. This truncated form may have been translocated via transposable element activity. Despite this, it exhibited a sharp, ~ fivefold increase in expression under salt stress, particularly in its long 3' UTR (Additional file 1: Fig. S10), suggesting a potential noncoding regulatory role in stress resistance (unpublished data). This functional specialization is further echoed by other B chromosome genes. Another B chromosome gene, *PaChr24B.82,* encodes a 259-amino acid protein that is partially homologous to *Arabidopsis GATA2* (67-nucleotide similarity), a transcriptional regulator involved in brassinosteroid and light signalling that promotes photomorphogenesis [68]. Interestingly, no orthologues of *PaChr24B.82* were detected in other plant species, suggesting an ancestral lineage-specific translocation to chromosome B and further evolution to acquire a unique function. *PaChr24B.262* encodes a DDE endonuclease homologue known to coordinate metal ions for catalytic activity, facilitating site-specific DNA cleavage and subsequent strand transfer [69]. This enzyme family may contribute to transposable element proliferation in *P. australis* [70]. Interestingly, DDE proteins have undergone neofunctionalization in plants, serving as key regulators of heading date in rice, underscoring their versatile roles in plant development [71].

Similar to its counterparts in other Poaceae species, the B chromosome in *P. australis* may also undergo transmission drive that facilitates non-Mendelian inheritance. In the invasive populations, two B-located genes *PaChr24B.218* and *PaChr24B.240*, both of which are homologues of the *Arabidopsis SCC3* gene required for monopolar kinetochore orientation, are upregulated across nearly all cell types. Previous studies have associated microtubule-associated proteins, which influence cell division, with chromosomal drive mechanisms [72]. Additionally, the co-occurrence of duplicated homologous genes on the B chromosome may reflect synergistic activity or dosage compensation in meiotic regulation, potentially contributing to the stability and persistence of B chromosomes in the invasive lineage.

## Conclusions

This study generated a high-resolution cell atlas of a monocot shoot system by integrating single-cell and spatial transcriptomic data, providing a valuable resource for studying plant development. It also advances our understanding of invasive genomics by enabling comparisons between invasive and non-invasive populations. We demonstrated that North American invasive populations of *P. australis* exhibit gene expression profiles that are distinct from those of European source populations across various cell types, suggesting that invasive populations may have adopted novel strategies to cope with environmental challenges such as hypoxia and drought. Although most tissues showed conserved responses enriched for processes associated with light deprivation and sucrose starvation, cell-type-specific adaptations were also evident: parenchyma specifically upregulated genes involved in plant-type cell wall organization, whereas epidermal cells upregulated hypoxia-response genes and downregulated genes linked with biotic defences.

Moreover, our analysis revealed a correlation between B chromosome copy number and invasiveness-related gene expression profiles. At this stage, the correlation is only suggestive and does not establish a causal role for B chromosomes in the evolution of invasiveness. Establishing causality will require functional validation, for example by overexpressing or knocking out key B chromosome-specific genes (such as *IMP-α3* or *SCC3*) in *P. australis* and assessing their impact on invasion-related traits such as growth rate, photosynthesis, and biomass. Complementary population-level genomic approaches, such as genome-wide association studies in larger cohorts, could further strengthen causal inference.

## Methods

### Tissue dissociation and preparation of single-cell suspensions

To generate a comprehensive single-cell transcriptomic atlas for *P. australis*, we selected six individuals from the EU (non-invasive) and NAint (invasive) populations, with three biological replicates in each group. These plants were grown in the common garden of Shandong University under natural conditions. Prior to single-cell protoplast preparation, the plants were kept at room temperature (25 °C) for several days, after which the rhizome shoots were collected. From each individual, we collected between 5 and 22 buds, which were then pooled into a Petri dish for each sample. The rhizome shoots were cut into 1–2 mm strips and added to tubes containing 10 ml of enzyme mixture consisting of 2% cellulase R10, 0.8% macerozyme R-10, 0.5% pectinase Y-23, 0.1% BSA, 20 mM MES, 20 mM KCl,10 mM CaCL_2_ and 0.55 M mannitol with shaking at 60 rpm for 1 h at 28 °C. The cells were then filtered through a 40 µm cell strainer to remove debris and other contaminants. Cell activity was detected via trypan blue staining and the cell concentration was measured via a haemocytometer and a light microscope. We proceeded with protoplast preparations that presented a viability greater than 70% and contained intact cell nuclei. Finally, the protoplasts were resuspended in 8% (w/v) mannitol solution in preparation for loading onto the chromium controller of the 10 × Genomics platform. Approximately 1.6 × 10^4^ isolated single cells and enzyme gel beads were packed into a single oil droplet for single-cell RNA-seq library construction.

### Chromium 10 × Genomics library and sequencing

The cells were loaded onto the 10 × Chromium Single Cell Platform (10 × Genomics) at a concentration of 700–1200 cells/µl (Single Cell 3′ library and Gel Bead Kit v.3) as described in the manufacturer’s protocol. The generation of gel beads in emulsions (GEMs), barcoding, GEM-RT clean-up, complementary DNA amplification and library construction were performed according to the manufacturer’s protocol. Qubit was used for library quantification before pooling. The final library pool was sequenced on the Illumina Nova 6000 instrument by Shanghai Personal bio (Shanghai, China) via the 150 bp paired-end reads strategy.

### Spatial transcriptomics sample preparation

The invasive sample NAint61 from individuals used in single-cell analysis was selected for spatial transcriptomics. Fresh shoots were rapidly frozen in an isopentane and liquid nitrogen bath, embedded in optical cutting temperature (OCT) compound and stored at −80 °C. Frozen sections were prepared at a thickness of 20 μm via a cryostat, followed by RNA extraction and quality control. Samples with an RNA integrity number (RIN) value of ≥ 6 were retained to ensure RNA integrity during freezing. The spatial transcriptomics slides (BMKMANU S3000) consisted of 6.8 × 6.8 mm capture areas containing 2,000,000 barcoded spots (2.5 μm diameter; 4.8 μm center-to-centre spacing) arranged in a hexagonal grid. Each spot carried primers with a 5′ attachment site, cleavage site, T7 promoter, partial Read 1 Illumina handle, unique spatial barcode, UMI, and poly(dT)VN sequence. The tissue sections were mounted onto BMKMANU S3000 Tissue Optimization and Gene Expression Slides and stored at − 80 °C until use. Tissue optimization was first performed to determine the optimal permeabilization time for library construction. For gene expression library preparation, tissue sections were subjected to methanol fixation, fluorescence-based cell segmentation, haematoxylin and eosin (H&E) staining, and bright-field imaging. Permeabilization was conducted according to the manufacturer’s protocol. The released mRNA molecules were captured by spatially barcoded probes and reverse transcribed into cDNA, which was subsequently collected, converted to double-stranded DNA, and PCR- amplified. The resulting cDNA underwent enzymatic fragmentation, end repair with A-tailing, magnetic bead-based fragment selection, adapter ligation, purification, and sample index PCR to generate standard next-generation sequencing (NGS) libraries. Libraries that passed quality control were sequenced on an Illumina NovaSeq 6000 platform via a paired-end 150 bp strategy.

### Sequencing and data preprocessing of scRNA data

Raw scRNA-seq reads were first demultiplexed to separate the six individual samples (EU60, EU78, EU620, NAint61, NAint113, and NAint191), and the corresponding barcodes were removed to ensure accurate sample assignment. Clean reads were subsequently aligned to the chromosome-level *P. australis* reference genome via the CellRanger pipeline v8.0 [73]. The reference genome sequence of *P. australis* (NCBI accession number: ASM4037322v1) was obtained from previous work and slightly modified for completeness [20]. Because the 25 chromosomes span from telomeres to telomeres, the genome was considered fully assembled: the remaining contigs were replaced with complete chloroplast and mitochondrial genomes to ensure accurate annotation of organellar transcripts. Genome annotation of the modified assembly was performed via liftoff v1.6.3 [74] on the basis of the original gene models. The mkref function with default settings in CellRanger was used to generate the species-specific reference, whereas the count function quantified UMIs per gene and barcode, producing raw count matrices for downstream analyses. To ensure data quality and minimize technical artifacts, the count matrices were filtered via DropletUtils v1.26.0 [75] and scrater v1.36.0 [76]. Genes expressed in fewer than 20 cells and cells with fewer than 500 detected genes were removed to eliminate low-complexity observations. Cells exhibiting > 10% mitochondrial gene expression or > 30% chloroplast gene expression were considered dying or stressed and were excluded from further analysis. High-quality single-cell datasets were processed via Seurat v5.2.1 [77] for normalization, integration and clustering. Gene expression data were normalized and variance-stabilized via sctransform v0.4.2 [78], which applies regularized negative binomial regression to reduce technical noise and preserve biological variation [79]. Potential doublets were identified and removed using DoubletFinder v2.0.6 [80] to improve cluster purity. By default, 3000 highly variable genes were selected for dimensional reduction and cell clustering. The clean data from all six samples were merged and subjected to PCA to capture major sources of transcriptional variability [81]. To correct for intersample batch effects and ensure accurate cross-sample integration, we applied the Harmony integration method implemented in the R package Harmony v1.2.3 [82]. Thirty-five principal components were selected for downstream analyses on the basis of the variance explained and inspection of the elbow plot, ensuring an optimal balance between signal and noise retention. Cell clustering was performed via the Louvain community detection algorithm, and the resolution parameter was optimized by testing multiple values; a final resolution of 0.8 provided the best balance between cluster granularity and biological interpretability. Dimensionality reduction and visualization were achieved via both UMAP and t-SNE with the parameter *min.dist* = 0.1 and *n.neighbors* = 50.

### Differential gene expression of scRNA-seq data

Differential gene expression analysis of the scRNA data between the invasive and non-invasive groups was conducted via DEseq2 v1.48.1 [83]. To enable pseudobulk-level analysis, raw read counts from all cells within each cluster were aggregated by sample, and the original gene-by-cell matrix was transformed to a gene-by-replicate matrix via muscat v 1.22.0 [84]. The aggregated count data were normalized via the regularized log (rlog) transformation implemented in DEseq2, and PCA was performed to assess sample relationships. Differential expression was then evaluated between the two groups.

Because intragroup variation explained the majority of the variance, the initial analysis included three biological replicates per group. Since the two outlier samples exhibited a significant departure of mean reads per cell compared with the other samples, we performed DEG analysis via the peudobulk method while keeping the mean reads per cell as a covariate in the DESeq2 analysis. For each cluster, after the genes with low expression, which had fewer than 10 genes in all six samples were removed, differential expression was tested using the Wald test. The resulting *p* values were adjusted for multiple testing using the Benjamini‒Hochberg method to control the false discovery rate (FDR). Genes with an adjusted *p* value (FDR) < 0.05 and |log₂-fold change|> 2 were considered significantly differentially expressed. The log fold changes were further shrunk via the lfcShrink() function using the apeglm method [85] in DESeq2 to provide more accurate effect size estimates.

### Data analysis of spatial transcriptomics

For the spatial transcriptomic data, we generated multiple subspot levels by aggregating gene counts from different numbers of spots. For example, subspot level 1 represents data from a single spot, whereas subspot level 7 aggregates data from 127 spots to form one subspot. We initially evaluated all subspot levels to identify cell types. For downstream analyses, we selected subspot level 4 (with a spatial resolution of 20 µm) as the analysis unit, since the median number of expressed genes per cell at this level most closely matched that of the single-cell data. Additionally, we examined an alternative approach using image-segmented cells as analysis units, yielding a total of 78,448 identified cells. The quality of the sequencing data from Read 2 was tested using fastqc v 0.10.1, and the reads were aligned to the reference genome using STAR aligner v 2.7.11b and quantified via BSTMatrix v2.3. Cell segmentation was performed using BSTMatrix, which employs Cellpose v2.0, which is based on high-resolution images obtained during tissue sectioning. The resulting expression matrix was imported into R via the script CreateBmkObject.R, retaining features expressed in at least five cells and cells expressing at least 100 genes (parameters: min.cells = 5, min.features = 100). The data were subsequently processed in Seurat, normalized using SCTransform v2, and subjected to downstream analysis including PCA, neighbour finding, clustering, and UMAP visualization. Clustering was performed with a resolution parameter of 0.5, and spatial cluster-specific marker genes were identified using Seurat’s FindMarkers function.

### Cell type annotation and identification of gene markers for single-cell clusters

In total, 19 clusters were identified from the single-cell transcriptomic dataset. The marker genes for each cluster were subsequently identified using the FindAllMarkers function in Seurat. We set logfc.threshold = 1 to include only genes with at least a twofold change in expression, and min.pct = 0.1 to consider genes expressed in at least 10% of cells in either cluster. Only genes with positive fold changes (only.pos = TRUE) were retained, with a focus on markers that were upregulated in specific clusters. The ROC test (test.use = "roc") was applied to evaluate each gene’s ability to discriminate a given cluster from all others. The identified markers were subsequently ranked within each cluster in descending order of their power metric using the arrange function in dplyr v1.1.4 [86]. To elucidate the biological functions associated with each cluster, we examined the GO terms of the corresponding marker genes. For GO enrichment analysis, *P. australis* genes were first mapped to their *Arabidopsis thaliana* orthologuesvia BLAST v2.16.0 + with a best-hit approach under an E-value threshold of 10⁻^4^ [87]. The *Arabidopsis* genome assembly and GO annotations were sourced from TAIR (https://www.arabidopsis.org/). GO enrichment was then performed with Goatools v1.5.2 [88], employing all annotated genes as the background set and the differentially expressed genes as the study set. Enriched GO terms were identified using a significance threshold of *p* < 0.05 after FDR correction. The resulting enrichment profiles were visualized with clusterProfiler v 4.16.0 [89]. To validate the annotation accuracy of the cell types, we projected the cells and clusters of the single-cell dataset to the spatial transcriptomic slides via both Seurat and Tangram v1.0.4 [90]. Tangram was said to be one of the most accurate software tools for the projection thus far, as evidenced by a benchmark study [91]. We performed eight mapping experiments using single-cell data from individuals EU620 (six experiments) and NAint61 (two experiments), the latter being from the same individual as the spatial data. These experiments assessed mapping accuracy by using different gene sets as training features and calculating the correlation between the actual and predicted spatial expression. We exhaustively tested the following eight combinations of training gene sets and mapping parameters: (1) All genes: The full set of 27,693 overlapping genes between the single-cell and spatial datasets. (2) EU620-HVG spot mapping: The top 3,000, 2,000, and 1,000 HVGs for spot-level mapping (spot diameter: 20 μm). (3) EU620 cellpose mapping: Three thousand HVGs for mapping onto spatial cells recognized by Cellpose. (4) EU620-marker-based cell mapping**:** Cell type markers as training genes for projection to a cell-split dataset. (5) NAint61-marker-based spot mapping: The NAint61 single-cell dataset with cell type markers for spot-level mapping (spot diameter: 20 μm). (6) NAint61-HVG spot mapping: The NAint61 single-cell dataset with the top 1,000 HVGs for spot-level mapping (spot diameter: 20 μm). To assess transcriptomic similarity among cell clusters, we applied the MuSiC v1.0.0 R package to construct hierarchical dendrograms [92]. Using music_basis, we obtained the cross-subject design matrix and mean relative abundance matrix for 19 clusters for each individual sample. Hierarchical clustering with complete linkage was performed on their log-transformed Euclidean distances, producing dendrograms that depict transcriptomic relationships among clusters.

### RNA velocity and trajectory analysis

Following preprocessing steps, the Seurat object of EU620 was converted to h5ad format using anndata v0.11.4 [93] for compatibility with Python-based analyses. The RNA velocity was first evaluated with Velocyte v0.17.17 [94] to generate loom files containing spliced and unspliced transcript counts. Subsequent RNA velocity analysis was performed using scvelo v0.3.3 [95], employing the dynamical model with default parameters. We used Scanpy to compute the diffusion pseudotime (DPT), selecting as the root the cell showing the lowest value on the second diffusion component. The resulting pseudotime values were then used, in combination with the pseudotime, velocity, and connectivity kernels, to infer terminal states using CellRank [49], with the meristematic cell cluster (cluster 14) defined as the initial cell population.

### Whole-genome sequencing

To evaluate the copy number of B chromosomes at the population level, we re-analysed the RAD-seq dataset of 88 individuals obtained from previously published work [15]. After the reads of low quality were filtered out using Trimmomatic v0.39 [96], the clean reads were mapped to the reference genome with repeats masked, and the ratios of the average number of reads mapped to the B chromosome and all other chromosomes were calculated. In addition, whole-genome sequences were obtained from nine individuals, four from European sources and five from North American invasive populations. The DNA was extracted via the CTAB method, and sequenced via the BGI DNB-seq platform. The WGS datasets were cleaned and mapped to the reference genome using bwa mem v0.7.17 [97]. For the alignment of genomic reads, first, PCR duplicates were removed via GATK v4.5.0.0 MarkDuplicates [98] and then input into deepvariant v1.9.0 [99], followed by GLnexus v1.4.1 [100] to call the SNPs. The number of reads mapped to the B chromosome and the other 24 chromosomes was calculated via bamcoverage v3.5.5 [101]. The differences in the read depth ratios between populations were tested via the Mann‒Whitney U test, with *p* < 0.05 considered statistically significant.

### Tests for selective sweeps

To account for the potential confounding effect of purifying selection, we performed a selective sweep analysis on the basis of the site frequency spectrum (SFS) of the derived alleles. To polarize polymorphic loci, we first aligned whole-genome sequences from five genetic lineages (accession numbers: SRR15440741, SRR15440740, SRR29606799, SRR33298956, SRR33298959) to the reference genome via bwa mem. Ancestral state reconstruction was carried out with angsd v0.940 [102] on the basis of the most common alleles among these individuals. Unfolded SFS for minor alleles were calculated for each chromosome and population. For selective sweep analysis, each chromosome was divided into chunks, and derived sites with zero frequency were excluded. The resulting SFS for each chromosome and population were used as inputs for SweepFinder2 [103], with 5 kb windows for visualization. A composite likelihood ratio greater than 10 was regarded as high, and the results were visualized via Manhattan plots in R.

### RNA-seq of leaf tissues

We harvested mature leaves from nine individuals grown in a common garden at Shandong University in June 2023 and used them for transcriptomic analysis, enabling a direct comparison of gene expression profiles. The genetic background was inferred from the published phylogeographic pattern [15]. Total RNA was extracted using the RNAprep Pure Plant Kit (Tiangen, Beijing, China) according to the instructions provided by the manufacturer. The RNA concentration and purity were measured using NanoDrop 2000 (Thermo Fisher Scientific, Wilmington, DE). RNA integrity was assessed using the RNA Nano 6000 Assay Kit of the Agilent Bioanalyzer 2100 system (Agilent Technologies, CA, USA). A total of 1 μg of RNA per sample was used as input material for the preparations of the RNA samples. Sequencing libraries were generated using the Hieff NGS Ultima Dual-mode mRNA Library Prep Kit for Illumina following the manufacturer’s recommendations and index codes were added to attribute sequences to each sample. The libraries were sequenced on an Illumina NovaSeq X Plus platform to generate 150 bp paired-end reads. The quality of the reads was checked using fastqc (https://www.bioinformatics.babraham.ac.uk/projects/fastqc/). Adapters were clipped, and low-quality bases (Phred score < 20) were removed from the reads in 4 bp sliding windows using Trimmomatic v0.39 [96]. Clean reads were mapped to the reference genome using STAR aligner v 2.7.11b [104]. After sorting using samtools v1.16.1 [105], the mapped reads were processed using stringtie v2.2.1 [106] to obtain the gene and transcript count matrix. We conducted PCA on differentially expressed genes among the treatment groups using DEseq2 v 1.48.1 following rlog transformation of gene counts, which represent the number of reads mapped to the reference genome [83].

Genes whose adjusted *P*-value was < 0.01 according to the Benjamini and Hochberg’s approach for controlling the false discovery rate and whose |log₂ fold change| was ≥ 2 according to DESeq2 were considered differentially expressed. GO enrichment analyses of genes in the context of GO categories were performed to determine whether certain functional categories are overrepresented or enriched within the background whole-genome gene sets. Here, we tested enriched GO terms for the differentially expressed genes (DEGs) using Goatools v1.5.2 [88], with the criterion of *P* < 0.05 with Bonferroni correction.

### Orthologues among species and transcription factor prediction

Eight outgroup species, including *Arabidopsis* (TAIR 11), rice IRGSP1.0 (https://rapdb.dna.affrc.go.jp/download/irgsp1.html) [107], *Populus trichocarpa* v4.1(Phytozome) [35], *Sorghum bicolor* v3.1.1 (Phytozome) [108], *Pisum sativum* [28, 109], *Solanum lycopersicum* ITAG4.0 (Phytozome) [110], maize B73 v5 and *Setaria viridis* V2 reference genomes at https://plants.ensembl.org/ [111] were included to identify orthologues between species using Orthofinder v2.5.5 [112]. Transcription factors of the proteins in the common reed genome were predicted from PlantRegMap [113]. The alignment between B chromosome sequences was performed using Cobalt [114] on the NCBI website (https://www.ncbi.nlm.nih.gov/tools/cobalt/re_cobalt.cgi), and the visualization of conserved amino acid homologues was conducted via weblogo (https://weblogo.berkeley.edu/logo.cgi) [115]. The structure of the homologous genes was visualized using GSDS v2.0 [116]. All software versions are listed in Supplementary Table S20.

## Supplementary Information

Additional file 1: Figures S1-S21.

Additional file 2: Tables S1-S20.

Additional file 3: Supplementary Note 1. An additional analysis was conducted by regressing out the outliers within each genetic group and retaining only two samples per group, illustrating potential differences in photosynthesis between invasive and native populations.

## Data Availability

All sequences generated in this study have been stored in the NCBI database. The single-cell transcriptomes of six samples and the spatial transcriptomic data were deposited in the Gene Expression Omnibus (GEO) database with the accession number GSE295468 [117]. The whole genome sequencing and RNA-seq data were deposited in the Sequence Read Archive (SRA) database under project number PRJNA1254975 [118]. The published RAD-seq data were obtained from the NCBI SRA database under project number PRJNA753984 [15, 119]. The scripts used for data analysis were deposited in Zenodo [120] and github [121] repositories.
