# Supplementary material for: Single-cell and spatial transcriptomics in Phragmites australis reveal the association of B chromosomes with plant invasiveness
Source: Genome Biol. 2026 Apr 22;27:184. doi: 10.1186/s13059-026-04079-x (PMC13235201; doi:10.1186/s13059-026-04079-x)
Supplement: Supplementary file 2 — Additional file 2: Tables S1-S20. [file 13059_2026_4079_MOESM2_ESM.pdf]

Supplementary materials

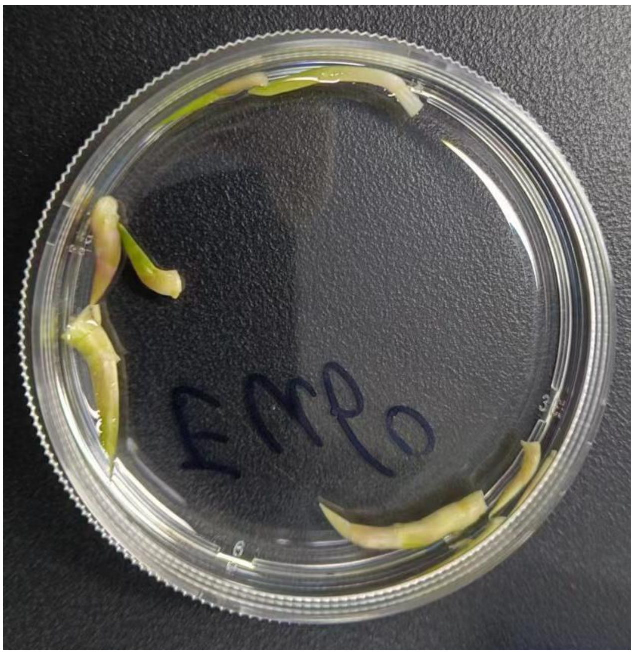

Fig. S1 Several shoots generated from rhizome of the same sample that were used to create single cell suspension for the individual EU60.

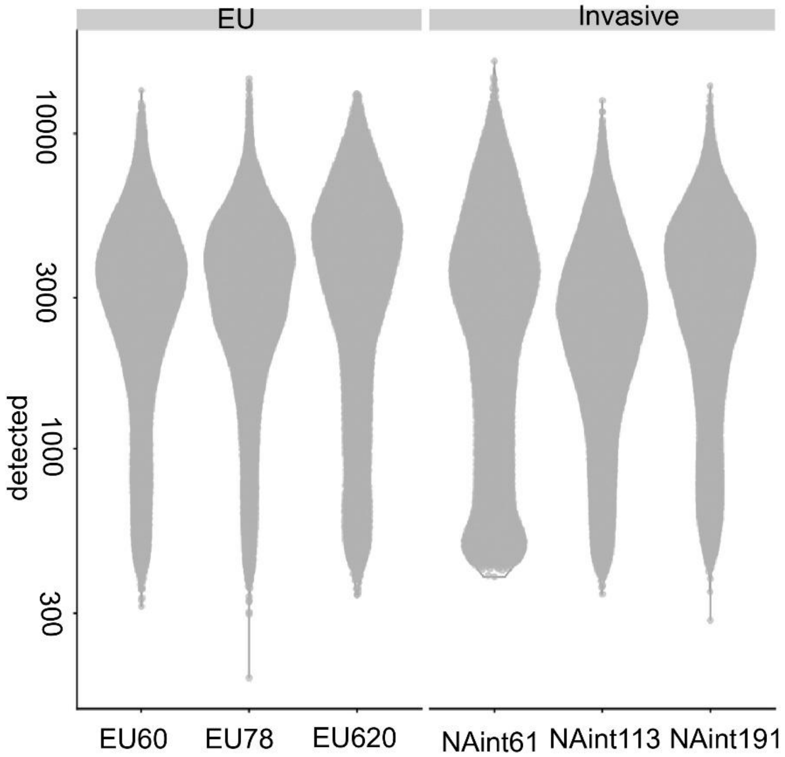

Fig. S2 Violin plot showing the distribution of detected genes per sample. Orange dots denote cells below the expression threshold (excluded during QC).

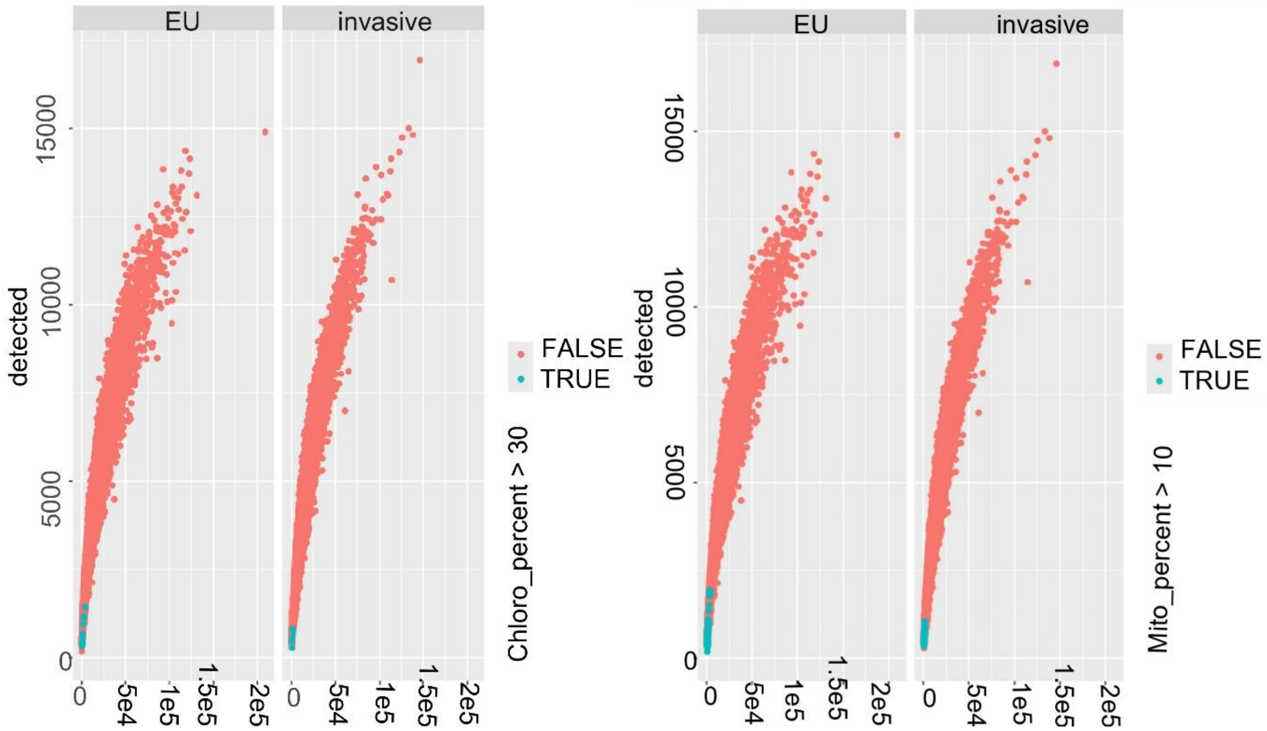

Fig. S3 Quality control of single-cell RNA-seq data: chloroplast and mitochondrial content across EU and invasive samples. Cells with more than 30% chloroplast transcripts (left panel) and more than 10% mitochondrial transcripts (right panel) are highlighted in blue, indicating potential low-quality or stressed cells that may be excluded from downstream analysis to improve data quality.

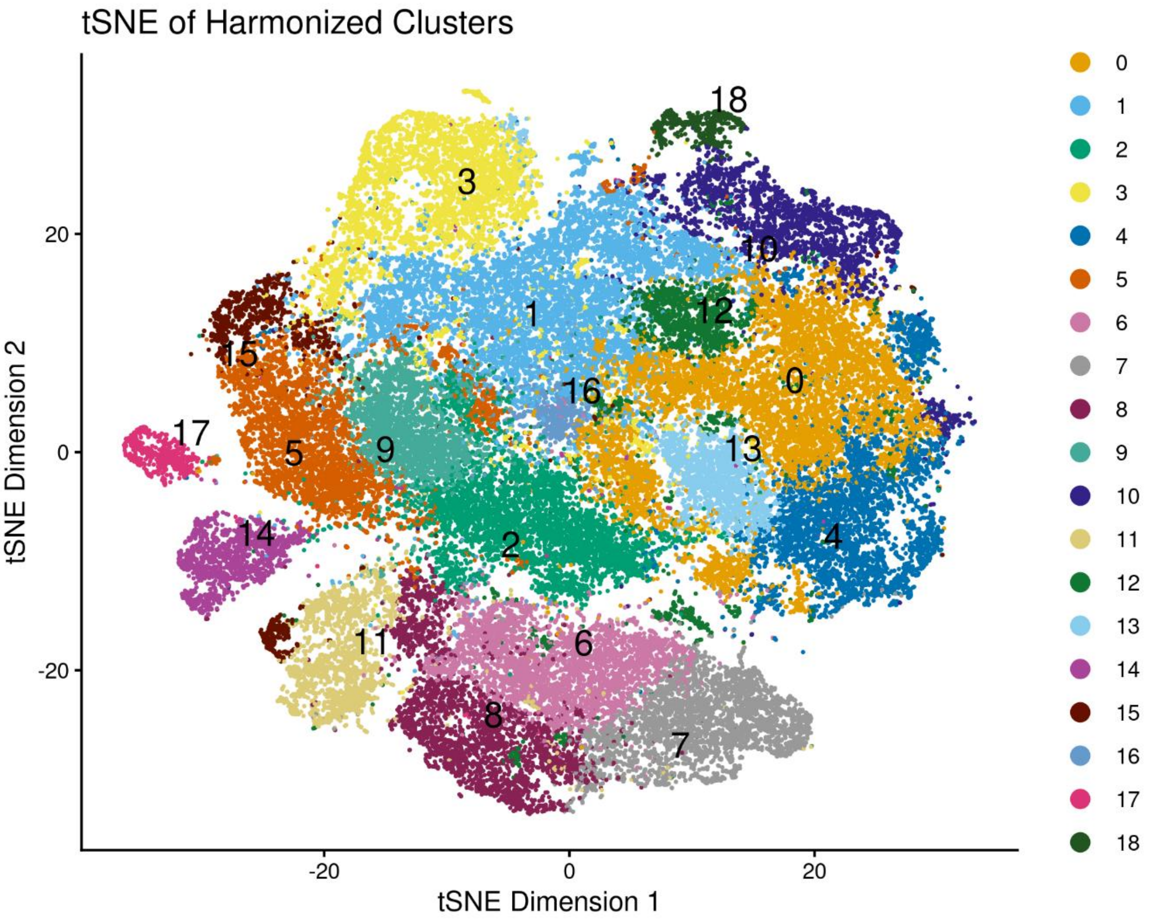

Fig. S4 The t-SNE projection of single-cell transcriptomic data clustered at a resolution of 0.8. Each dot represents an individual cell, colored by its assigned Seurat cluster (0–18).

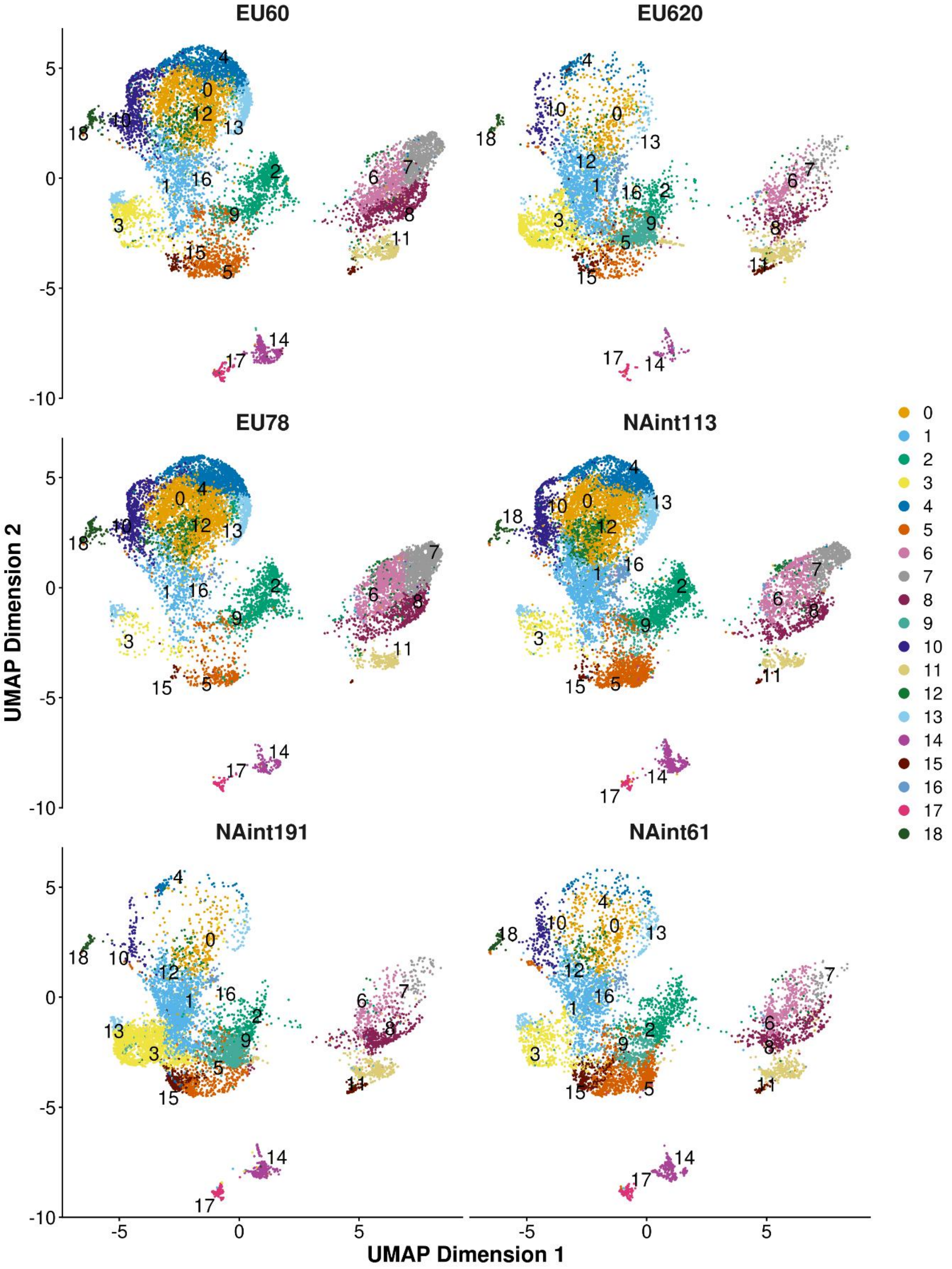

Fig. S5 UMAP projection of single-cell transcriptomic data, split by sample identity. Each panel represents a separate sample, with cells colored according to their respective UMAP coordinates. The samples are labeled on the x-axis, and the coordinates reflect the dimensionality reduction based on the top 30 principal

components of the data. The plots are arranged in a 2-column layout to facilitate comparison of UMAP embeddings across samples.

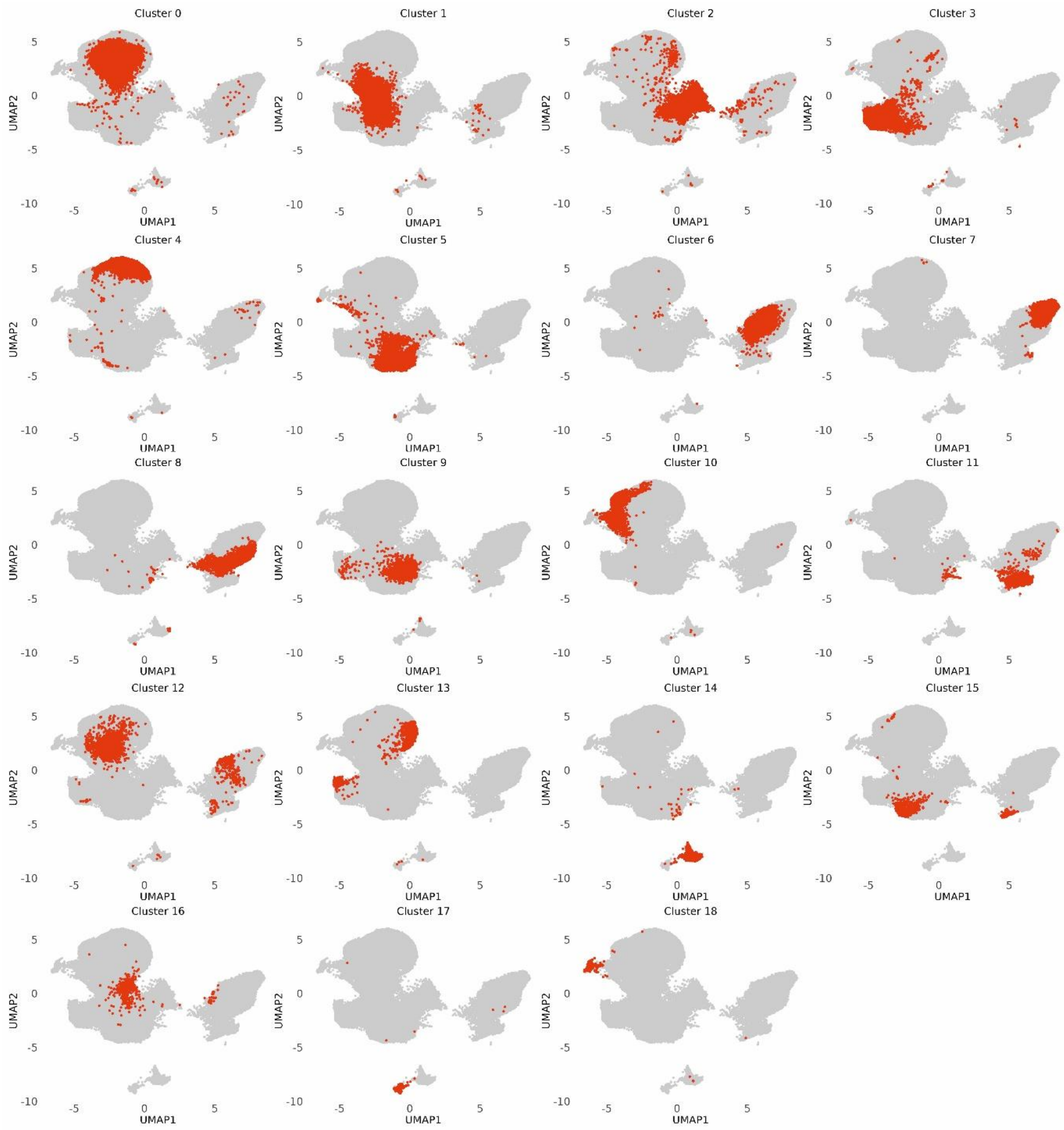

Fig. S6 UMAP visualization of the 19 identified single-cell clusters.

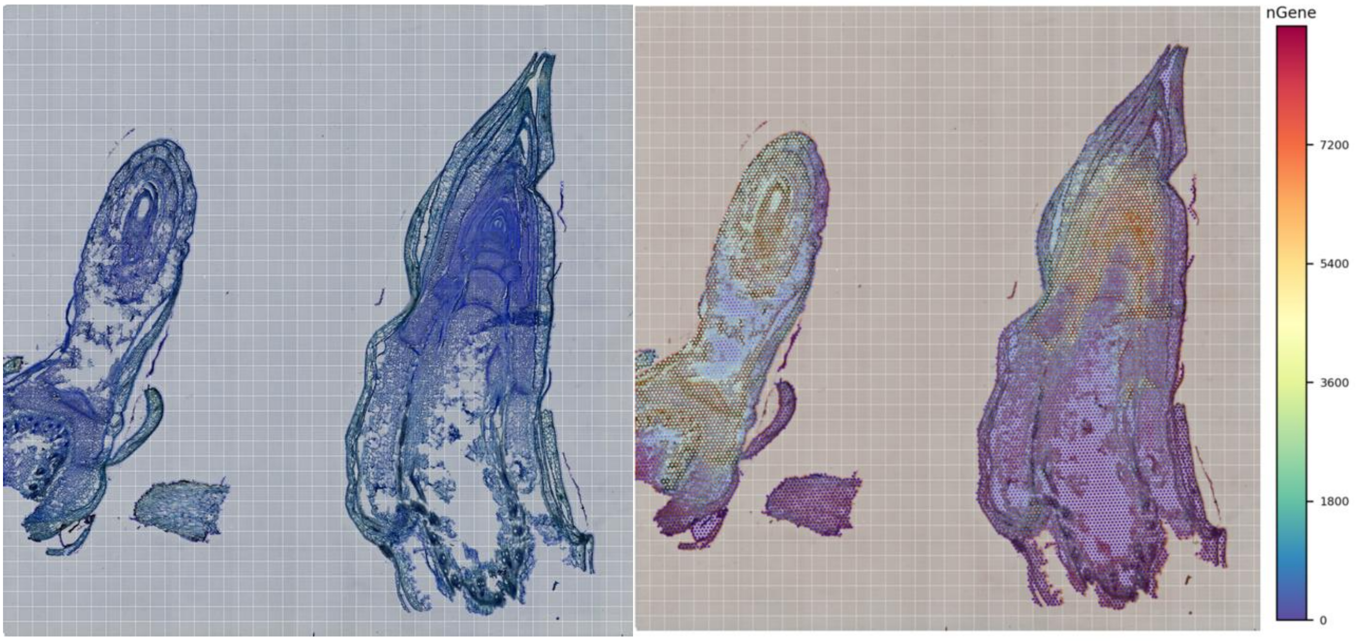

Fig. S7 Heatmap of gene expression levels in spatial transcriptomic tissues. The left panel displays the anatomical structure of the common reed bud used for spatial transcriptomics. The right panel shows spatial gene expression data superimposed on the histological tissue section, with each spot representing a capture location. A color gradient (blue to red) indicates the number of detected genes (nGene) per spot.

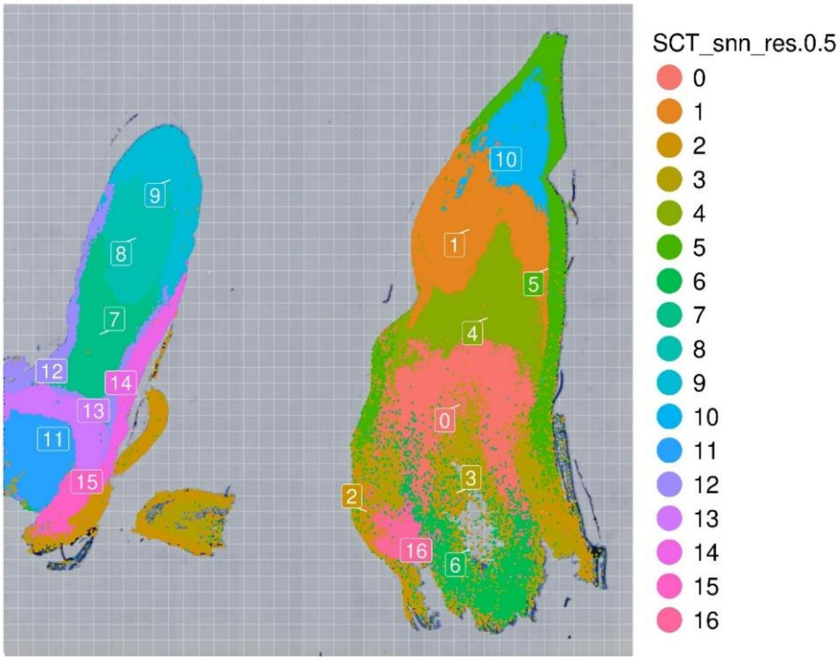

Fig. S8 Seventeen clusters identified from spatial transcriptomics data at a spatial

resolution of 20 μm, using level 4 subspot granularity. This clustering reveals the spatial organization of transcriptionally distinct regions within the tissue.

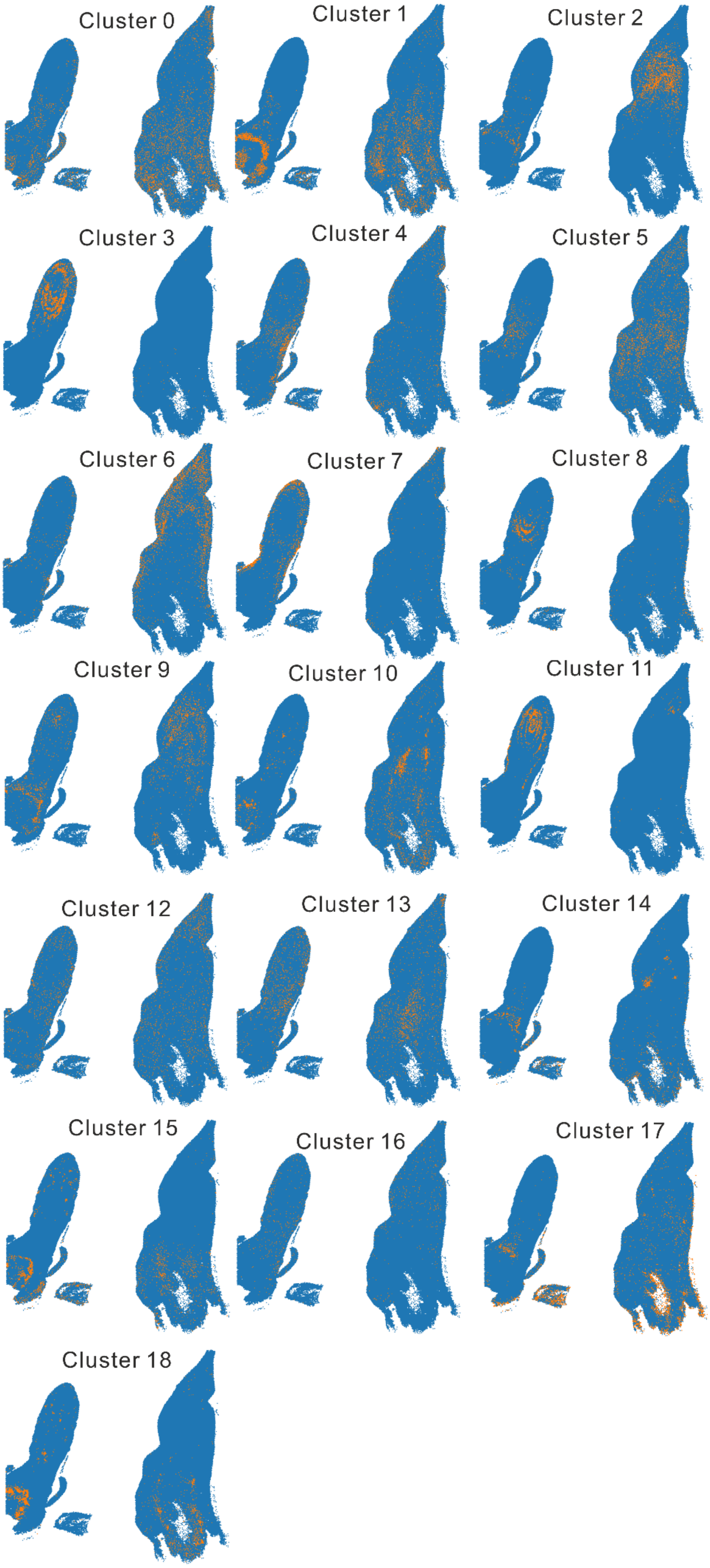

Fig. S9 Spatial projection of single-cell transcriptomic clusters onto the spatial transcriptomics dataset. Each mapped cell cluster reflects its inferred spatial localization within the tissue, enabling integration of single-cell resolution with spatial context. Orange dots indicate the predicted locations of the mapped cells on the tissue section.

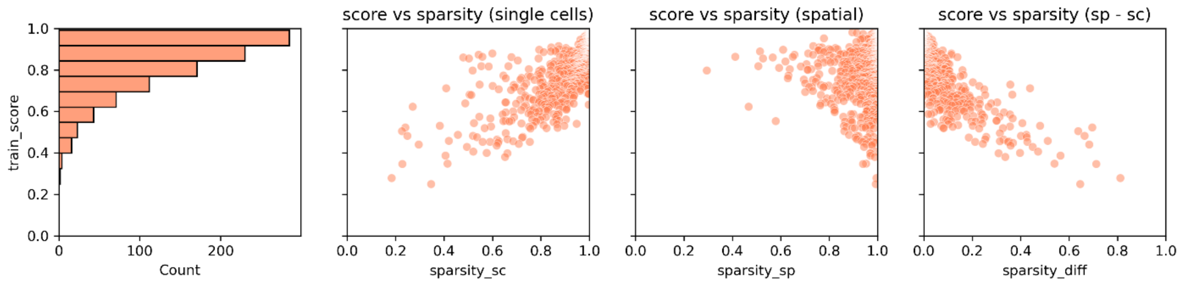

Fig. S10. Training scores and cell sparsity in spatial projections of single-cell data at 20 μm spot-level resolution. The figure shows, when using 1000 highly variable genes as training genes, the distribution of mapping scores during model training and the sparsity of predicted cell assignments across spatial spots, providing an assessment of projection accuracy and resolution.

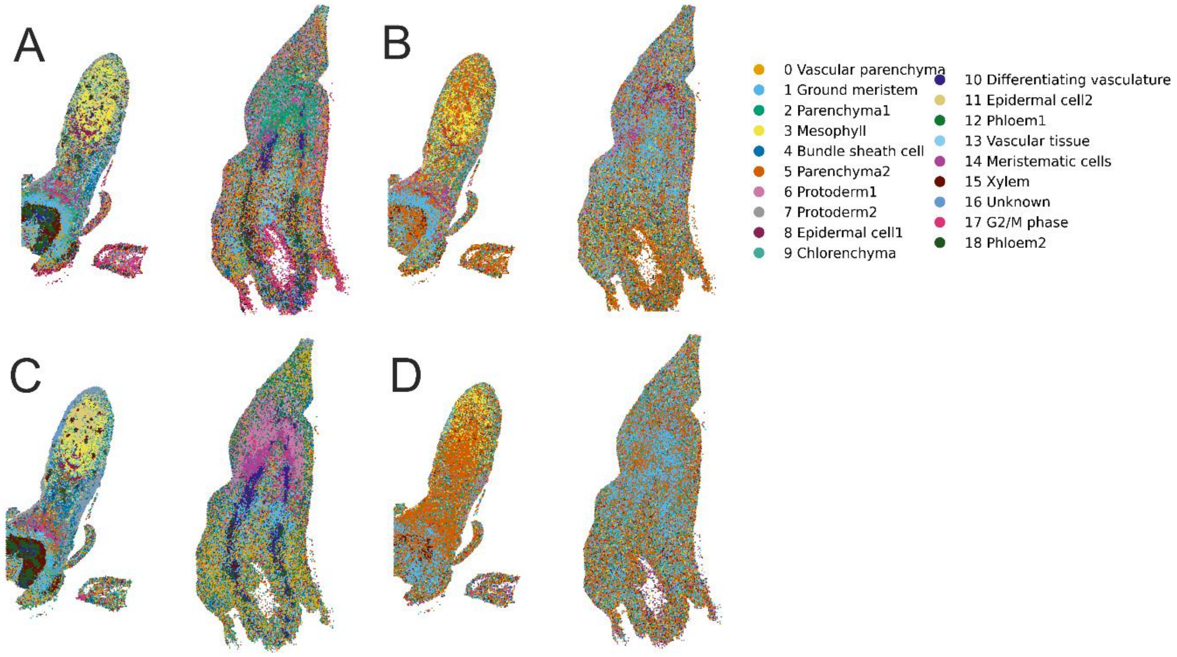

Fig. S11. Spatial projections of single-cell data at 20 μm spot-level resolution. (A) Projection trained on 1,000 highly variable genes, visualized in cluster mode. (B) Same projection as A, mapped in cell-to-space mode, achieving a high average mapping score of 0.825. (C) Projection trained on all transcriptomic genes mapped in cluster mode**.** (D) Same projection as (C), resulting in a low average mapping score of 0.252 but exhibiting superior visual coherence, mapped in cell to space mode.

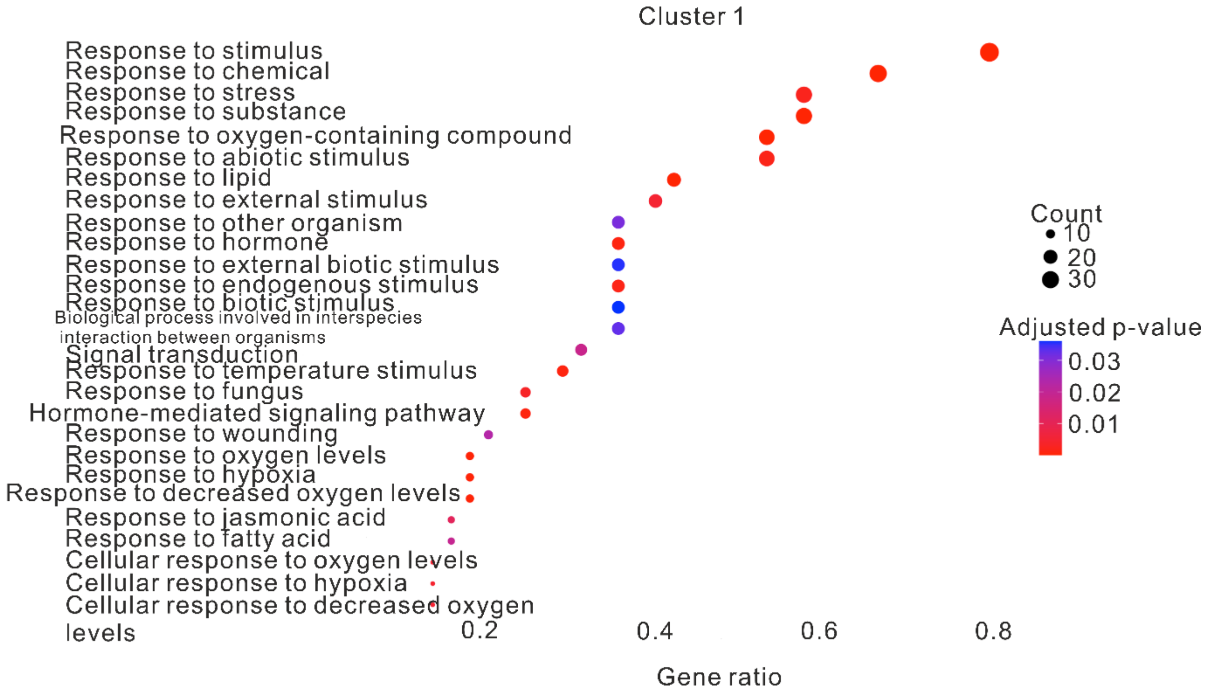
Cluster 1
Response to stimulus
Response to chemical
Response to stress
Response to substance
Response to oxygen-containing compound
Response to abiotic stimulus
Response to lipid
Response to external stimulus
Response to other organism
Response to hormone
Response to external biotic stimulus
Response to endogenous stimulus
Response to biotic stimulus
Biological process involved in interspecies interaction between organisms
Signal transduction
Response to temperature stimulus
Response to fungus
Hormone-mediated signaling pathway
Response to wounding
Response to oxygen levels
Response to hypoxia
Response to decreased oxygen levels
Response to jasmonic acid
Response to fatty acid
Cellular response to oxygen levels
Cellular response to hypoxia
Cellular response to decreased oxygen levels
0.2
0.4
0.6
0.8
Gene ratio
Count
10
20
30
Adjusted p-value
0.03
0.02
0.01

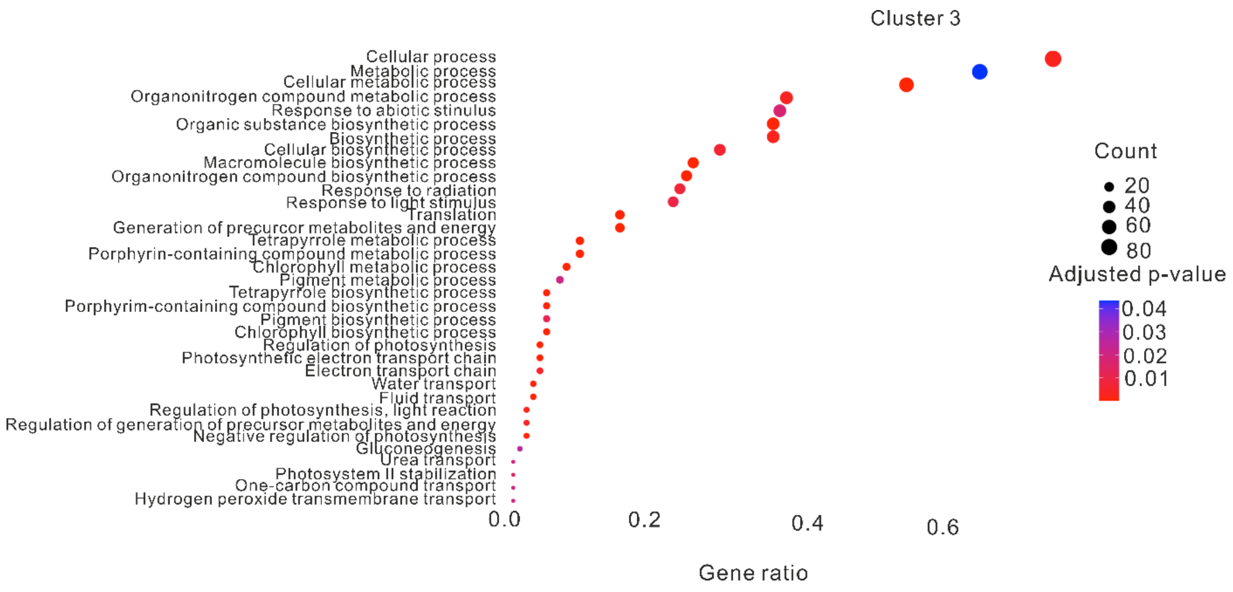
Cluster 3
Cellular process
Metabolic process
Cellular metabolic process
Organonitrogen compound metabolic process
Response to abiotic stinulus
Organic substance biosynthetic process
Biosynthetic process
Cellular biosynthetic process
Macromolecule biosynthetic process
Organonitrogen compound biosynthetic process
Response to radiation
Response to light stimulus
Translation
Generation of precurcor metabolites and energy
Tetrapyrrole metabolic process
Porphyrin-containing compound metabolic process
Chlorophyll metabolic process
Pigment metabolic process
Tetrapyrrole biosynthetic process
Porphyrim-containing compound biosynthetic process
Pigment biosynthetic process
Chlorophyll biosynthetic process
Regulation of photosynthesis
Photosynthetic electron transport chain
Electron transport chain
Water transport
Fluid transport
Regulation of photosynthesis, light reaction
Regulation of generation of precursor metabolites and energy
Negative regulation of photosynthesis
Gluconeogenesis
Urea transport
Photosystem II stabilization
One-carbon compound transport
Hydrogen peroxide transmembrane transport
0.0
0.2
0.4
0.6
Gene ratio
Count
20
40
60
80
Adjusted p-value
0.04
0.03
0.02
0.01

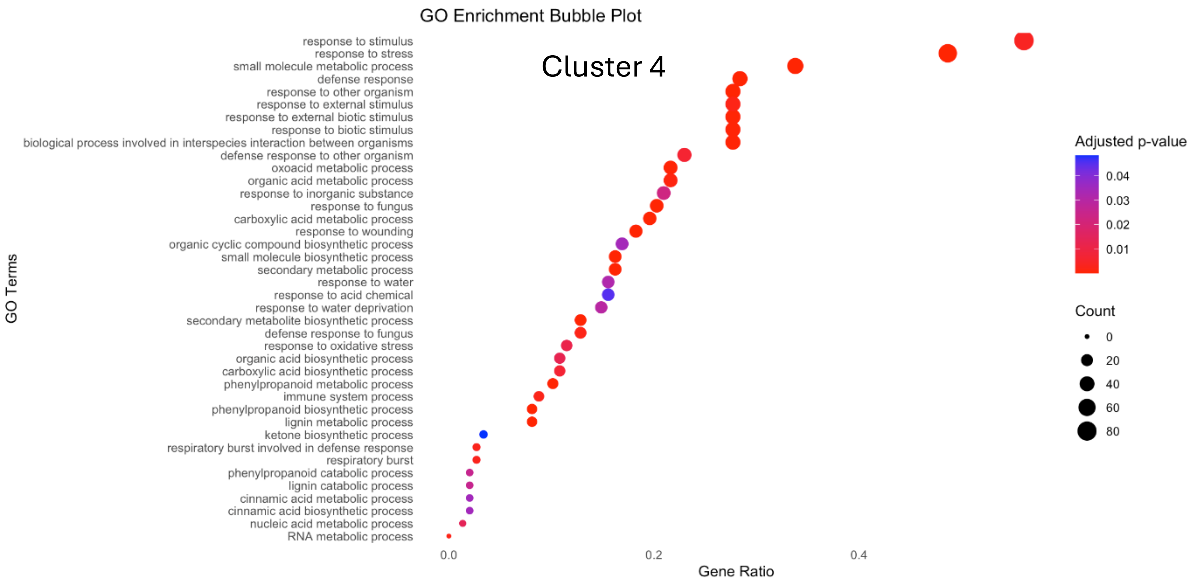
GO Enrichment Bubble Plot
Cluster 4
response to stimulus
response to stress
small molecule metabolic process
defense response
response to other organism
response to external stimulus
response to external biotic stimulus
response to biotic stimulus
biological process involved in interspecies interaction between organisms
defense response to other organism
oxoacid metabolic process
organic acid metabolic process
response to inorganic substance
response to fungus
carboxylic acid metabolic process
response to wounding
organic cyclic compound biosynthetic process
small molecule biosynthetic process
secondary metabolic process
response to water
response to acid chemical
response to water deprivation
secondary metabolite biosynthetic process
defense response to fungus
response to oxidative stress
organic acid biosynthetic process
carboxylic acid biosynthetic process
phenylpropanoid metabolic process
immune system process
phenylpropanoid biosynthetic process
lignin metabolic process
ketone biosynthetic process
respiratory burst involved in defense response
respiratory burst
phenylpropanoid catabolic process
lignin catabolic process
cinnamic acid metabolic process
cinnamic acid biosynthetic process
nucleic acid metabolic process
RNA metabolic process
GO Terms
0.0
0.2
0.4
Gene Ratio
Adjusted p-value
0.04
0.03
0.02
0.01
Count
0
20
40
60
80

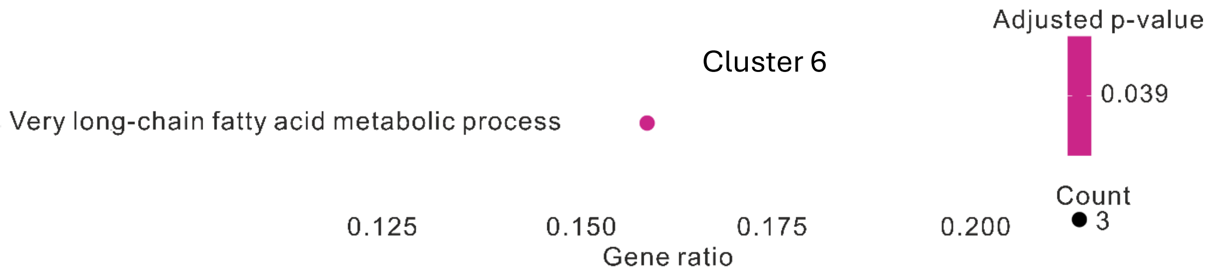
Cluster 6
Very long-chain fatty acid metabolic process
0.125
0.150
0.175
0.200
Gene ratio
Adjusted p-value
0.039
Count
3

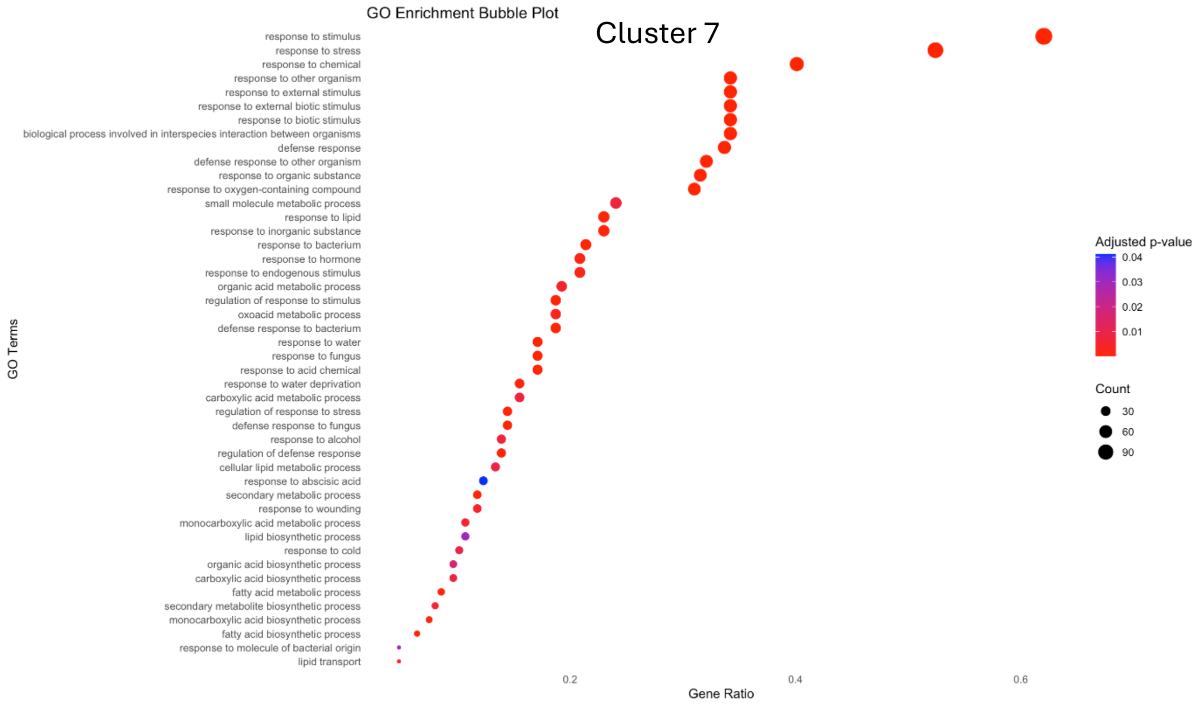
GO Enrichment Bubble Plot
Cluster 7
response to stimulus
response to stress
response to chemical
response to other organism
response to external stimulus
response to external biotic stimulus
response to biotic stimulus
biological process involved in interspecies interaction between organisms
defense response
defense response to other organism
response to organic substance
response to oxygen-containing compound
small molecule metabolic process
response to lipid
response to inorganic substance
response to bacterium
response to hormone
response to endogenous stimulus
organic acid metabolic process
regulation of response to stimulus
oxoacid metabolic process
defense response to bacterium
response to water
response to fungus
response to acid chemical
response to water deprivation
carboxylic acid metabolic process
regulation of response to stress
defense response to fungus
response to alcohol
regulation of defense response
cellular lipid metabolic process
response to abscisic acid
secondary metabolic process
response to wounding
monocarboxylic acid metabolic process
lipid biosynthetic process
response to cold
organic acid biosynthetic process
carboxylic acid biosynthetic process
fatty acid metabolic process
secondary metabolite biosynthetic process
monocarboxylic acid biosynthetic process
fatty acid biosynthetic process
response to molecule of bacterial origin
lipid transport
GO Terms
0.2
0.4
0.6
Gene Ratio
Adjusted p-value
0.04
0.03
0.02
0.01
Count
30
60
90

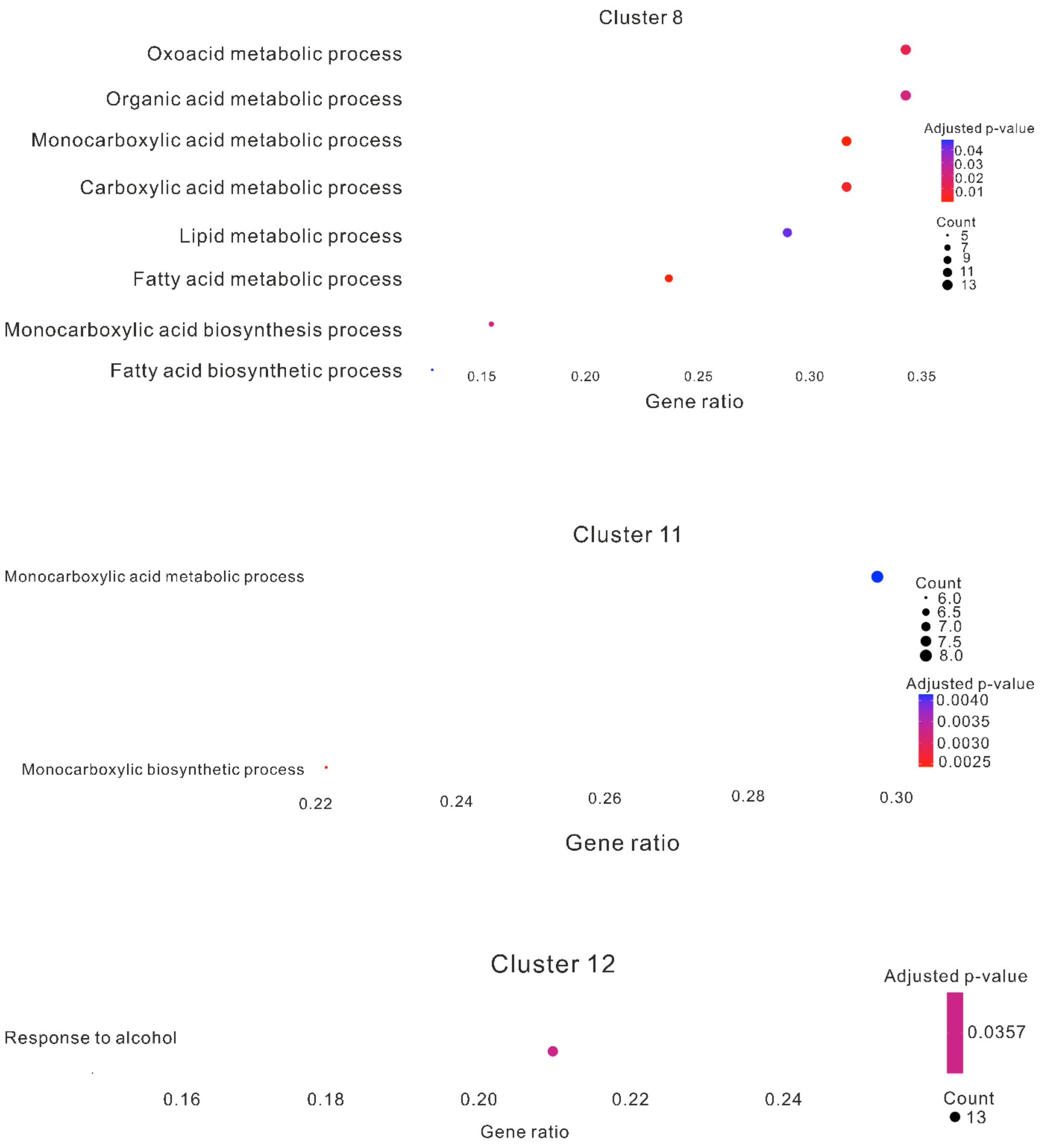

Cluster 8
Oxoacid metabolic process
Organic acid metabolic process
Monocarboxylic acid metabolic process
Carboxylic acid metabolic process
Lipid metabolic process
Fatty acid metabolic process
Monocarboxylic acid biosynthesis process
Fatty acid biosynthetic process
0.15
0.20
0.25
0.30
0.35
Gene ratio
Adjusted p-value
0.04
0.03
0.02
0.01
Count
5
7
9
11
13
Cluster 11
Monocarboxylic acid metabolic process
Monocarboxylic biosynthetic process
0.22
0.24
0.26
0.28
0.30
Gene ratio
Count
6.0
6.5
7.0
7.5
8.0
Adjusted p-value
0.0040
0.0035
0.0030
0.0025
Cluster 12
Response to alcohol
0.16
0.18
0.20
0.22
0.24
Gene ratio
Adjusted p-value
0.0357
Count
13

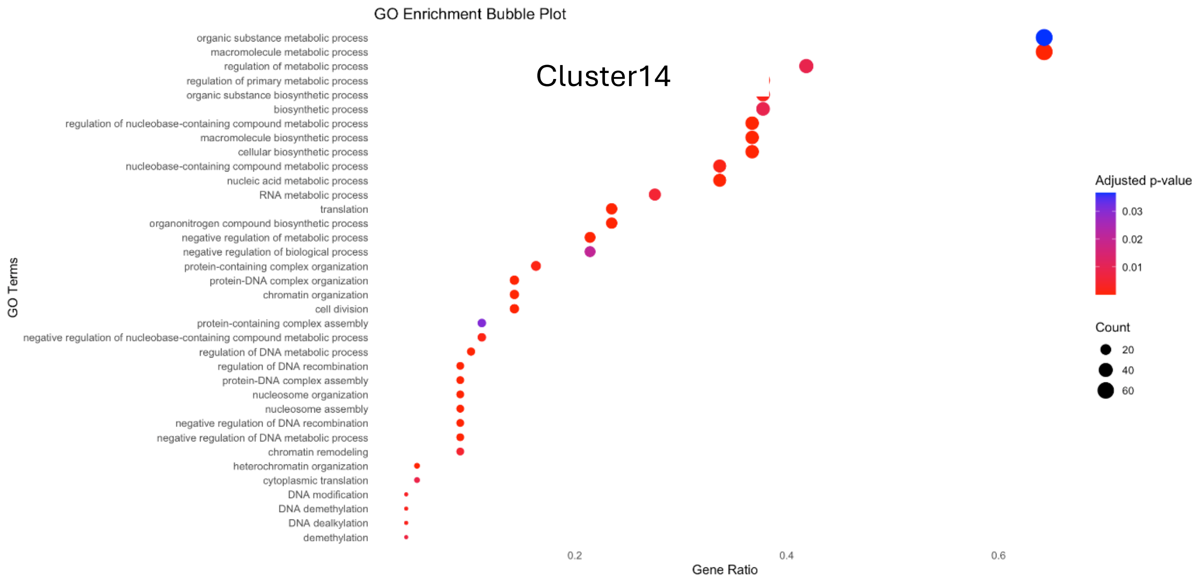

GO Enrichment Bubble Plot
Cluster14
organic substance metabolic process
macromolecule metabolic process
regulation of metabolic process
regulation of primary metabolic process
organic substance biosynthetic process
biosynthetic process
regulation of nucleobase-containing compound metabolic process
macromolecule biosynthetic process
cellular biosynthetic process
nucleobase-containing compound metabolic process
nucleic acid metabolic process
RNA metabolic process
translation
organonitrogen compound biosynthetic process
negative regulation of metabolic process
negative regulation of biological process
protein-containing complex organization
protein-DNA complex organization
chromatin organization
cell division
protein-containing complex assembly
negative regulation of nucleobase-containing compound metabolic process
regulation of DNA metabolic process
regulation of DNA recombination
protein-DNA complex assembly
nucleosome organization
nucleosome assembly
negative regulation of DNA recombination
negative regulation of DNA metabolic process
chromatin remodeling
heterochromatin organization
cytoplasmic translation
DNA modification
DNA demethylation
DNA dealkylation
demethylation
GO Terms
0.2
0.4
0.6
Gene Ratio
Adjusted p-value
0.03
0.02
0.01
Count
20
40
60

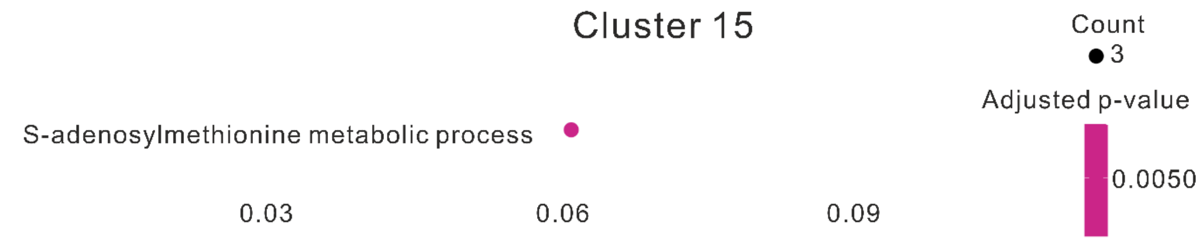

Cluster 15
Count
3
Adjusted p-value
0.0050
S-adenosylmethionine metabolic process
0.03
0.06
0.09

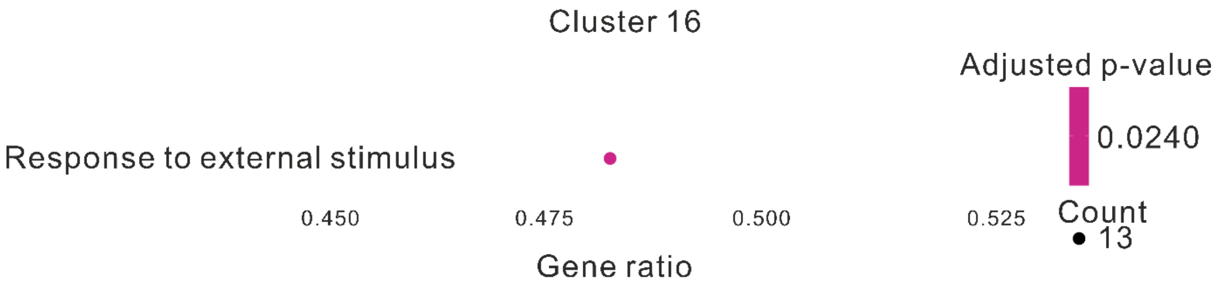

Cluster 16
Adjusted p-value
0.0240
Response to external stimulus
0.450
0.475
0.500
0.525
Count
13
Gene ratio

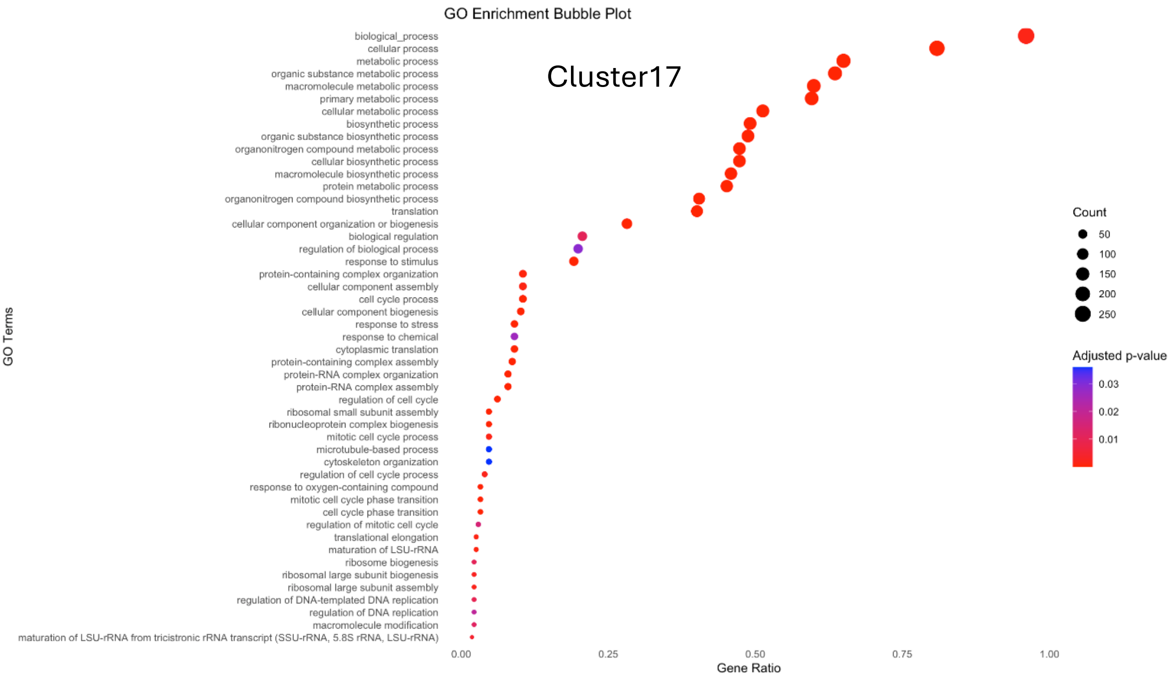

Fig. S12 Significantly enriched Gene Ontology (GO) terms identified for each cluster. These annotations highlight the predominant biological processes associated with the gene expression profiles of each cluster.

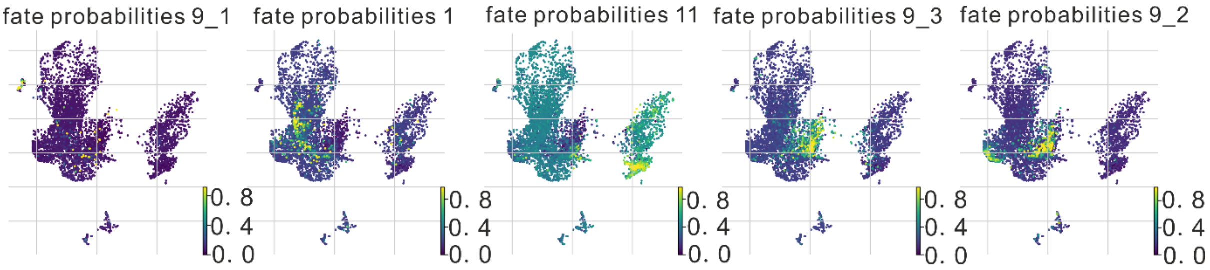

Fig. S13 Inferred fate probability of sample EU620 following dpt pseudotime trajectory.

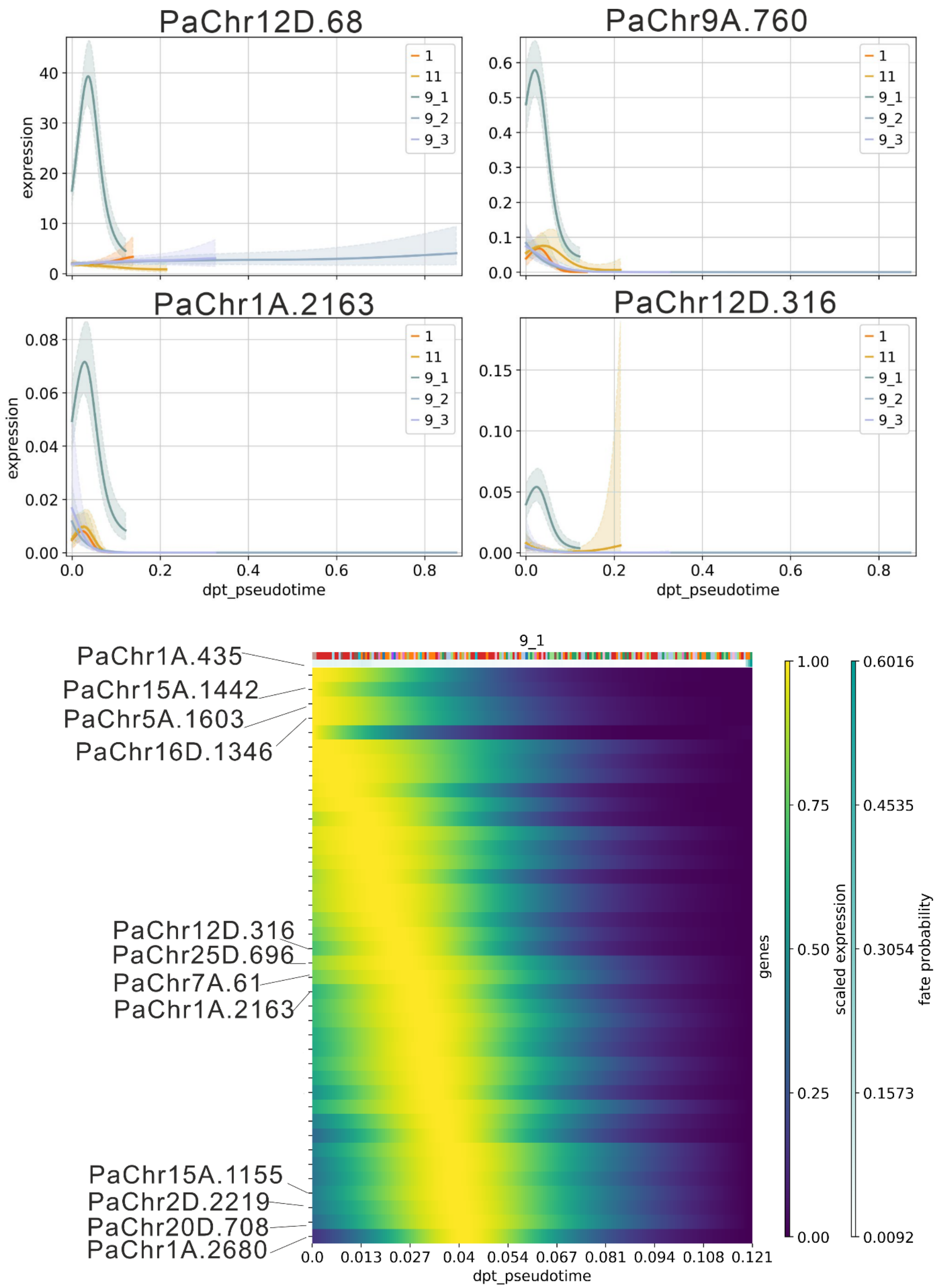
PaChr12D.68
PaChr9A.760
PaChr1A.2163
PaChr12D.316
expression
dpt_pseudotime
1
11
9_1
9_2
9_3
PaChr1A.435
PaChr15A.1442
PaChr5A.1603
PaChr16D.1346
PaChr12D.316
PaChr25D.696
PaChr7A.61
PaChr1A.2163
PaChr15A.1155
PaChr2D.2219
PaChr20D.708
PaChr1A.2680
genes
scaled expression
fate probability
0.0 0.013 0.027 0.04 0.054 0.067 0.081 0.094 0.108 0.121
0.6016 0.4535 0.3054 0.1573 0.0092
1.00 0.75 0.50 0.25 0.00

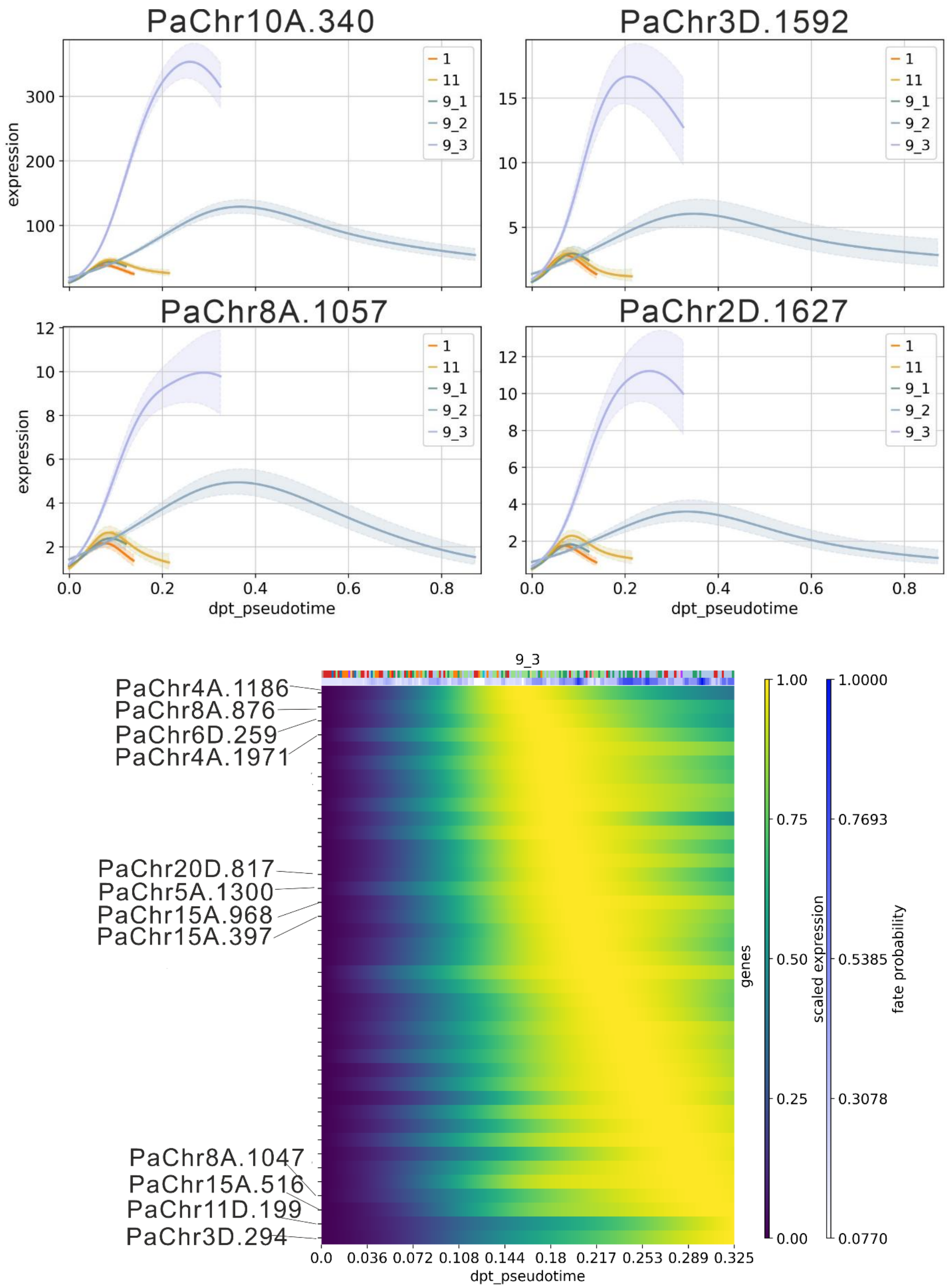

Fig. S14 Driver genes and gene expression levels across different states of Cluster 9 along the pseudotime trajectory. Panel a,b shows cell state 1, 3 respectively.

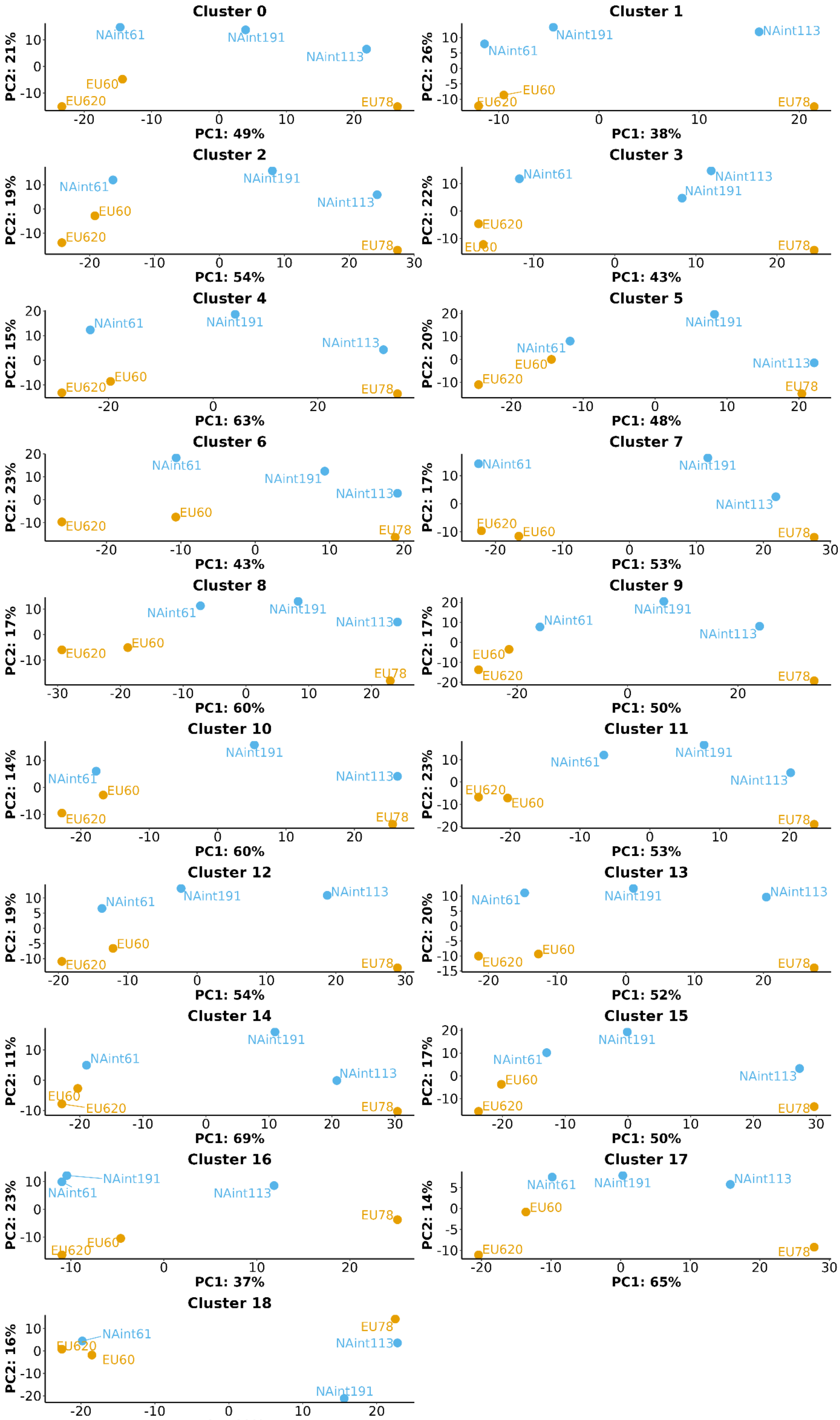
Cluster 0
NAint61
NAint191
NAint113
EU60
EU620
EU78
PC2: 21%
PC1: 49%
Cluster 1
NAint191
NAint113
NAint61
EU60
EU620
EU78
PC2: 26%
PC1: 38%
Cluster 2
NAint61
NAint191
NAint113
EU60
EU620
EU78
PC2: 19%
PC1: 54%
Cluster 3
NAint61
NAint113
NAint191
EU620
EU60
EU78
PC2: 22%
PC1: 43%
Cluster 4
NAint61
NAint191
NAint113
EU60
EU620
EU78
PC2: 15%
PC1: 63%
Cluster 5
NAint191
NAint61
EU60
EU620
NAint113
EU78
PC2: 20%
PC1: 48%
Cluster 6
NAint61
NAint191
NAint113
EU60
EU620
EU78
PC2: 23%
PC1: 43%
Cluster 7
NAint61
NAint191
NAint113
EU620
EU60
EU78
PC2: 17%
PC1: 53%
Cluster 8
NAint61
NAint191
NAint113
EU60
EU620
EU78
PC2: 17%
PC1: 60%
Cluster 9
NAint61
NAint191
NAint113
EU60
EU620
EU78
PC2: 17%
PC1: 50%
Cluster 10
NAint191
NAint61
NAint113
EU60
EU620
EU78
PC2: 14%
PC1: 60%
Cluster 11
NAint61
NAint191
NAint113
EU620
EU60
EU78
PC2: 23%
PC1: 53%
Cluster 12
NAint61
NAint191
NAint113
EU60
EU620
EU78
PC2: 19%
PC1: 54%
Cluster 13
NAint61
NAint191
NAint113
EU60
EU620
EU78
PC2: 20%
PC1: 52%
Cluster 14
NAint61
NAint191
NAint113
EU60
EU620
EU78
PC2: 11%
PC1: 69%
Cluster 15
NAint61
NAint191
NAint113
EU60
EU620
EU78
PC2: 17%
PC1: 50%
Cluster 16
NAint191
NAint61
NAint113
EU78
EU60
EU620
PC2: 23%
PC1: 37%
Cluster 17
NAint61
NAint191
NAint113
EU60
EU620
EU78
PC2: 14%
PC1: 65%
Cluster 18
NAint61
EU620
EU60
EU78
NAint113
NAint191
PC2: 16%

Fig. S15 Principal component analysis (PCA) of pseudobulk expression profiles for each cluster (A) in the single-cell dataset. PCA normalization accounted for the experimental variable 'group' and the covariate 'mean reads per cell'. In each cluster, invasive and ancestral European populations were separated along PC2, while PC1 captured a large proportion of gene expression variability within populations.

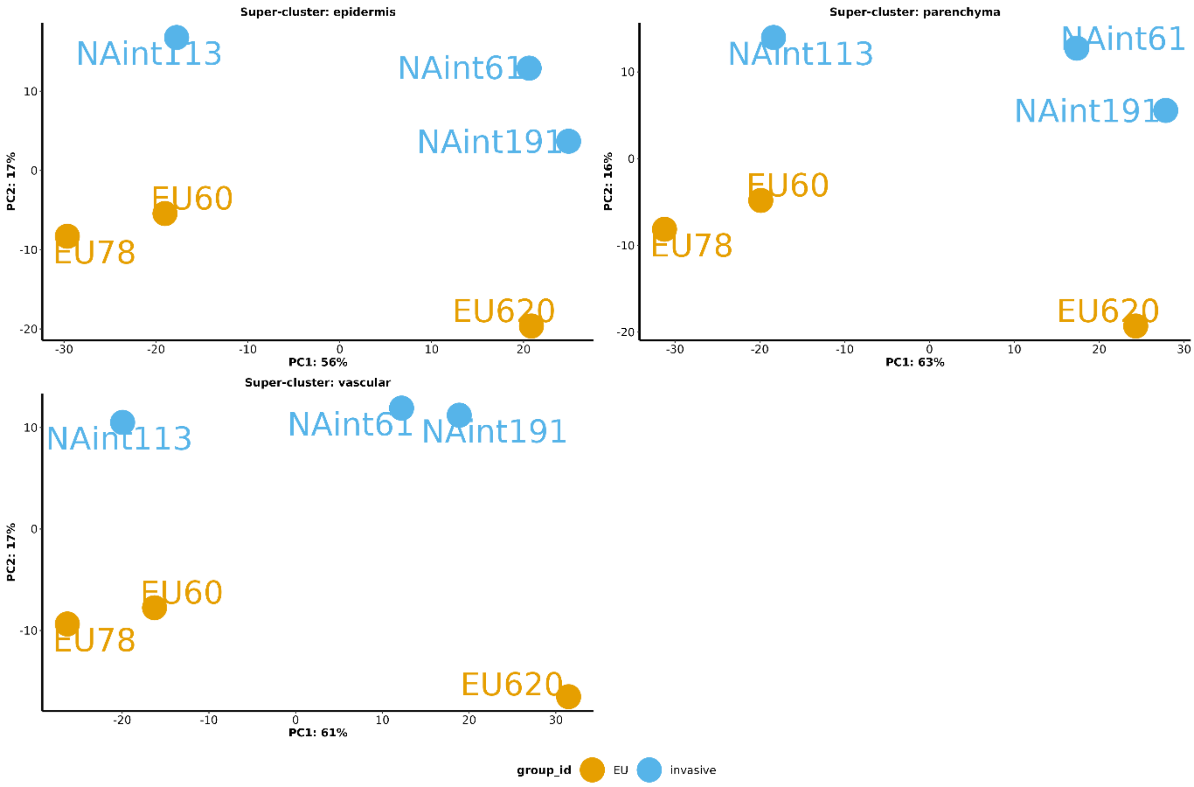

Fig. S16 Principal component analysis (PCA) of pseudobulk expression profiles for each supercluster in the single-cell dataset. PCA normalization accounted for the experimental variable 'group' and the covariate 'mean reads per cell'.

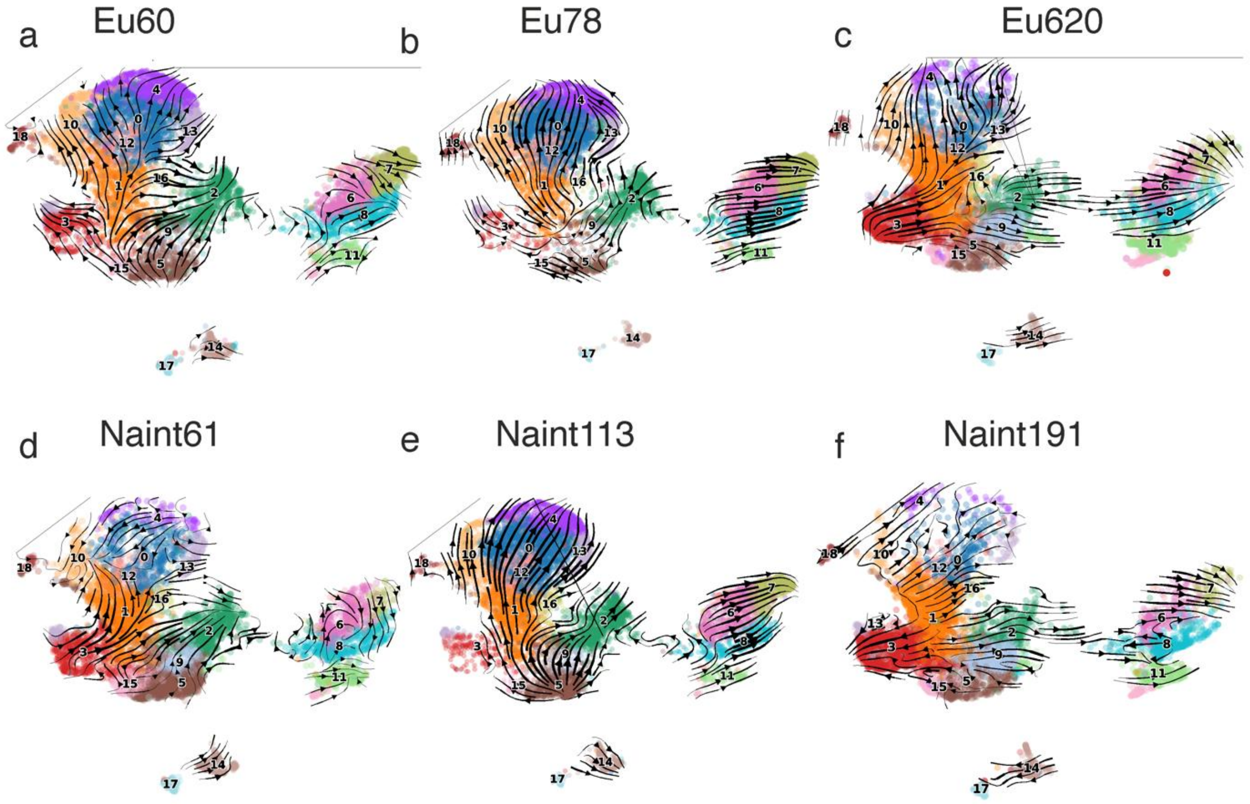

Fig. S17 RNA velocity analysis for each sample, depicting transient transcriptional dynamics within the shoot system. Arrows represent the inferred direction and magnitude of RNA flow, indicating the potential future states of individual cells and highlighting developmental trajectories across distinct tissue types.

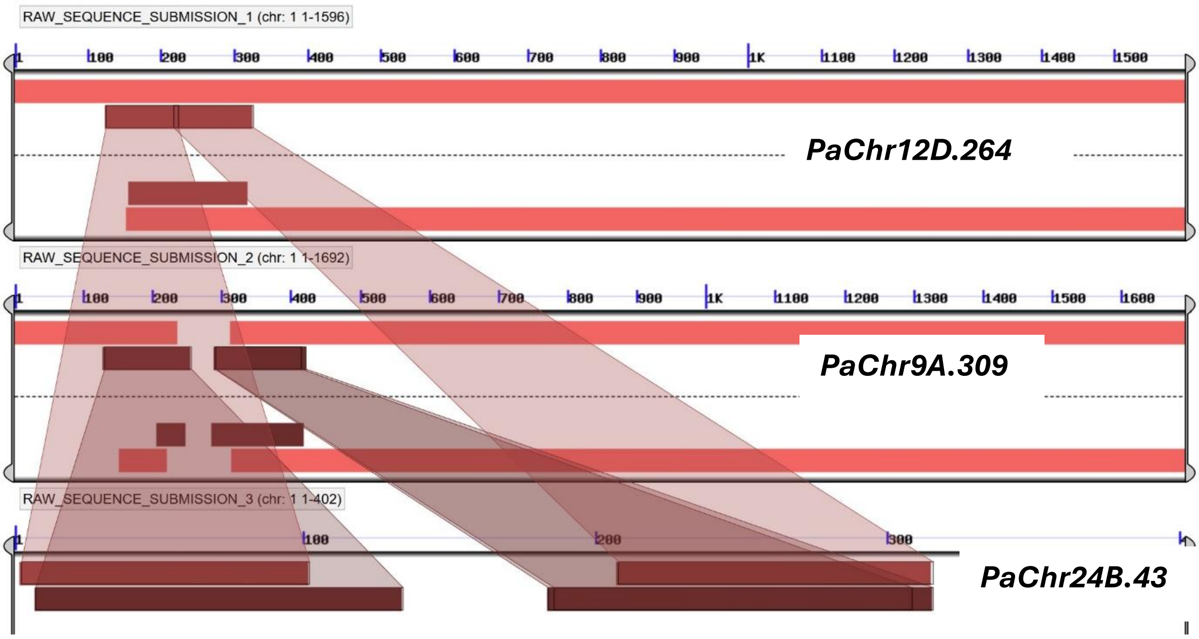

Fig. S18 Alignment comparison of B chromosome gene *PaChr24B.43* (third track) with *PaChr12D.264* (first track) and *PaChr9A.309* (second track). Analysis was performed using CoGe.

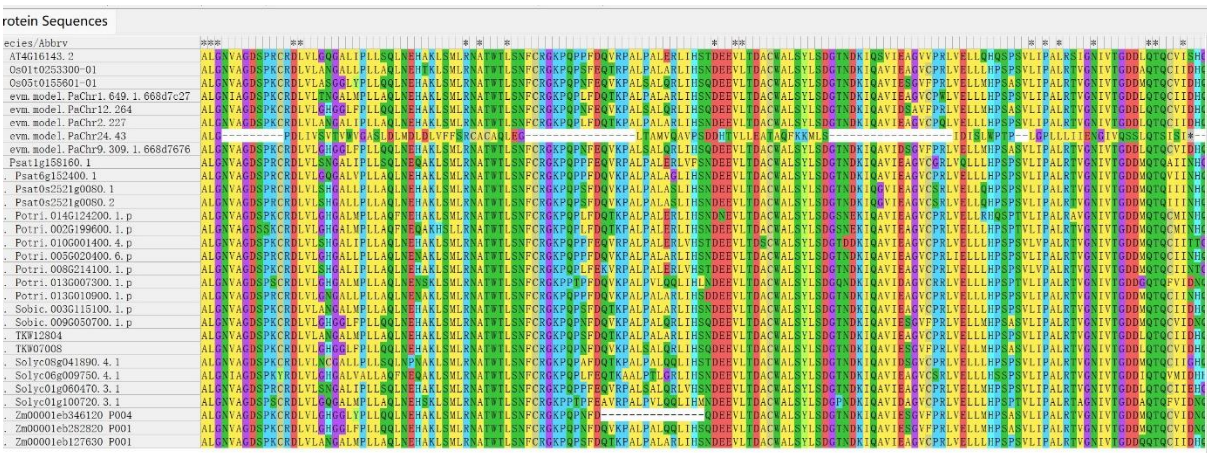

Fig. S19 Protein sequence alignment of the *P. australis* B chromosome gene *PaChr24B.43* with its homologs. The conserved regions are characterized by a high rate of sequence divergence from other closely related species.

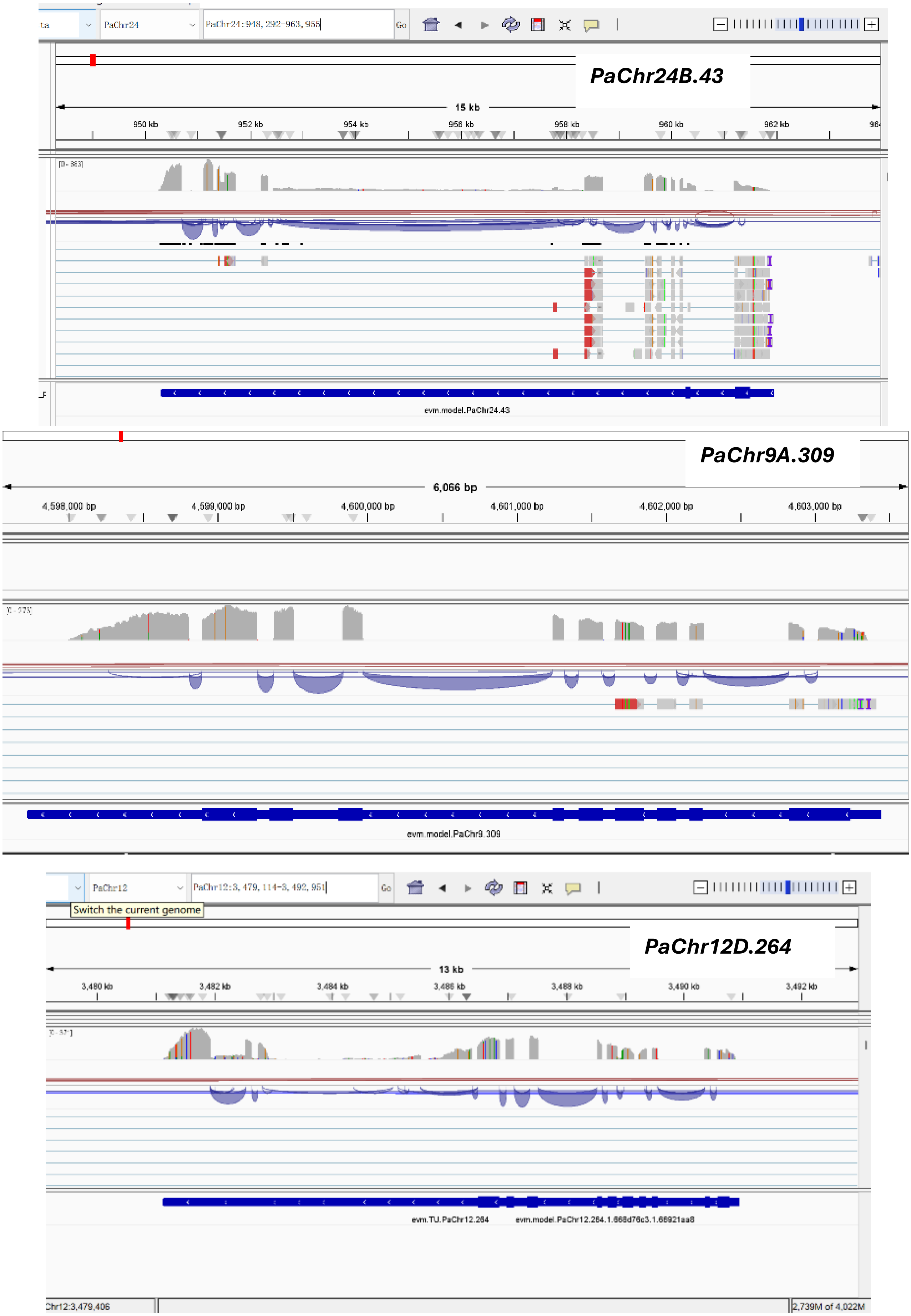

Fig. S20. IGV visualization illustrates the homologous gene structure of *PaChr24B.43*, *PaChr9A.309* and *PaChr12D.264*. The grep track displays bulk RNA-seq data from an invasive North American individual, mapped to this B chromosome gene.

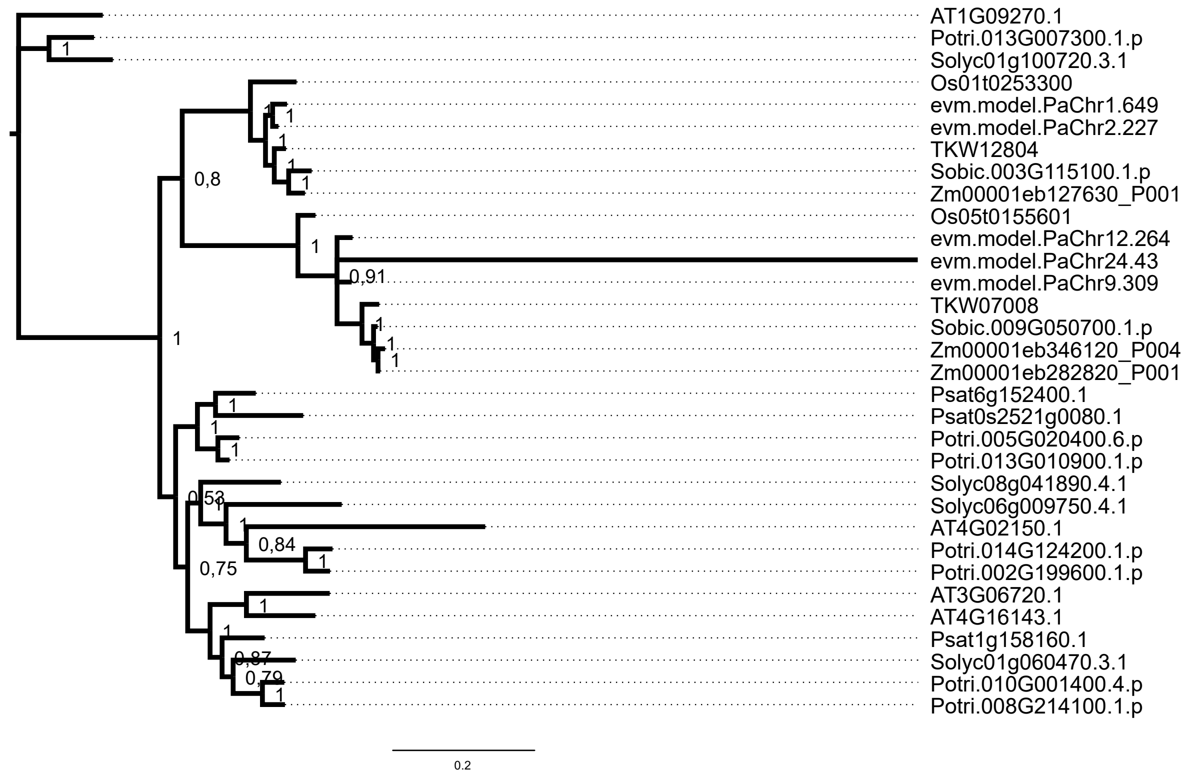

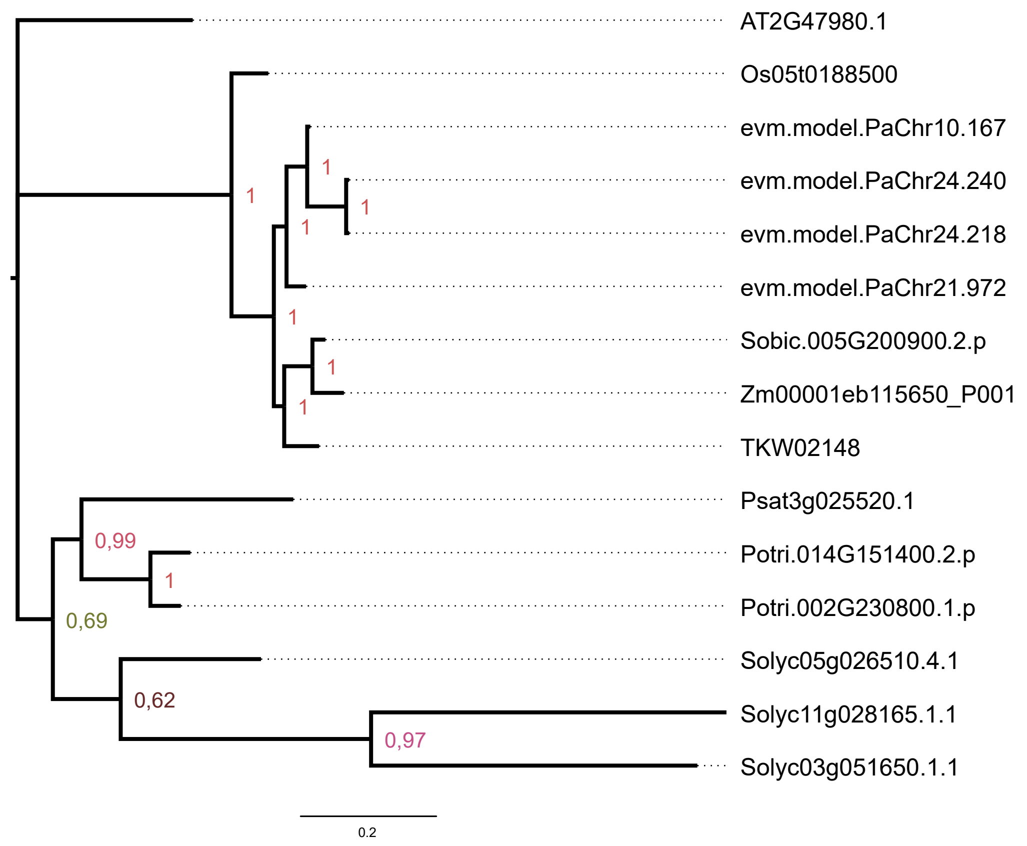

Fig. S21 Phylogeny of three genes (*PaChr24B.43*, *PaChr24B.240*, *PaChrB.218*) from the B chromosome, Illustrating the origin of B chromosome genes.
