# Supplementary material for: Single-cell and spatial transcriptomics in Phragmites australis reveal the association of B chromosomes with plant invasiveness
Source: Genome Biol. 2026 Apr 22;27:184. doi: 10.1186/s13059-026-04079-x (PMC13235201; doi:10.1186/s13059-026-04079-x)
Supplement: Supplementary file 3 — Additional file 3: Supplementary Note 1. An additional analysis was conducted by regressing out the outliers within each genetic group and retaining only two samples per group, illustrating potential differences in photosynthesis between invasive and native populations. [file 13059_2026_4079_MOESM3_ESM.pdf]

Supplementary Note 1

Concerning the high within group variability, we tentatively excluded outlier samples (EU620 and Naint113) and retained the two most consistent replicates from each population for further hypothesis exploration. Among these, with an adjusted p-value cutoff of 0.05 and | Log_2FC | > 2, the vascular tissue exhibited the highest number of DEGs, with 749 genes upregulated and 728 genes downregulated in the invasive individuals. In the epidermis, 671 genes were upregulated and 697 were downregulated. The parenchyma displayed fewer DEGs, with 283 genes upregulated and 373 downregulated. Across all five tissue types, 45 genes were commonly upregulated in the invasive individuals. These genes were significantly enriched for GO terms related to photosynthetic light harvesting and the regulation of precursor metabolite and energy generation. In contrast, 27 genes were consistently downregulated across all tissues, but no significant GO term enrichment was identified for this group. For the meristematic cell cluster, among the 442 DEGs, 279 genes were upregulated in the invasive lineage, while 163 genes were downregulated. The upregulated genes were primarily enriched in processes related to light harvesting in photosynthesis, translation, chlorophyll biosynthesis, response to abiotic stimuli (including radiation, cold, high and low light intensity), photosynthetic electron transport chain, plastid translation, granum assembly, and membrane bending. On the other

hand, the downregulated genes were enriched in processes related to stress response, defense response, and lipid transport.

Excluding mesophyll cells which showed the fewest DEGs between groups, a total of 102 genes were commonly upregulated across the remaining four tissues, enriched in the light reaction of photosynthesis, including processes such as response to light stimulus, light harvesting in photosystem I and II, photosystem II assembly and stabilization, and the photosynthetic electron transport chain. Ninety-four genes were downregulated, with enrichment observed in respiratory burst involved in the defense response. The largest number of shared DEGs were detected between the epidermis and vascular systems, with 344 upregulated genes enriched in processes related to photosynthesis, stress responses (including hypoxia, low light intensity, heat, and cold), and brassinosteroid and glucosinolate metabolic processes. Specifically, the invasive population upregulated 36 genes in response to hypoxia, 31 genes in response to heat, 26 genes in response to cold, and 20 genes in response to light intensity. Additionally, 385 downregulated genes were enriched in immune system processes, including defense responses to bacteria and fungi, respiratory burst in defense, response to wounding, and various hormonal responses (abscisic acid, salicylic acid), as well as processes related to leaf senescence.

The upregulated genes exclusively in epidermis was enriched in

processes related to response to brassinosteroids, fatty acids, jasmonic acid, oxidative stress, and endogenous stimuli. Biosynthetic processes such as flavonoid biosynthesis and monocarboxylic acid biosynthesis, as well as metabolic processes like xyloglucan metabolism, carboxylic acid metabolism, and olefinic compound metabolism, were also uniquely enriched in the epidermis. In contrast, the vascular tissue exhibited specific enrichment in response to mannose, response to hydrogen peroxide, cellular response to sulfur starvation, and cellular response to chemical stimulus.

One gene, *SCC3*, located on the B chromosome, was consistently upregulated across all three clusters. In cluster 14, most of the upregulated DEGs are primarily involved in photosystem functioning (*PSAG*, *PSAH2*, *PSAN*, *PSBP1*, *LHCB6*, *PSBW*, *LHCB4.2*, *PSBQA*, *LHB1B2*), chloroplast accumulation (*CPN60A*), chloroplast protein translation (*PRPL34*), and plant hormone response (*XTH24*, *PYL11*). In cluster 11 (epidermal cell), the top upregulated genes are primarily involved in anthocyanin biosynthesis (*ANS*, *CHS*, *DFR*). In cluster 18, the upregulated genes encode proteins involved in environmental stimuli responses (*TCH4*, *SUS4*, *PYL11*).
